# Therapeutic functions of astrocytes to treat α-synuclein pathology in Parkinson’s disease

**DOI:** 10.1073/pnas.2110746119

**Published:** 2022-07-15

**Authors:** Yunseon Yang, Jae-Jin Song, Yu Ree Choi, Seong-hoon Kim, Min-Jong Seok, Noviana Wulansari, Wahyu Handoko Wibowo Darsono, Oh-Chan Kwon, Mi-Yoon Chang, Sang Myun Park, Sang-Hun Lee

**Affiliations:** ^a^Department of Biochemistry and Molecular Biology, College of Medicine, Hanyang University, Seoul 04763, Korea;; ^b^Hanyang Biomedical Research Institute, Hanyang University, Seoul 04763, Korea;; ^c^Graduate School of Biomedical Science and Engineering, Hanyang University, Seoul 04763, Korea;; ^d^Department of Pharmacology, Ajou University School of Medicine, Suwon 16499, Korea;; ^e^Center for Convergence Research of Neurological Disorders, Ajou University School of Medicine, Suwon 16499, Korea

**Keywords:** astrocyte, α-synuclein, Parkinson’s disease, proteostasis, transplantation

## Abstract

Intraneuronal inclusions of misfolded α-synuclein (α-syn) and prion-like spread of the pathologic α-syn contribute to progressive neuronal death in Parkinson’s disease (PD). Despite the pathologic significance, no efficient therapeutic intervention targeting α-synucleinopathy has been developed. In this study, we provide evidence that astrocytes, especially those cultured from the ventral midbrain (VM), show therapeutic potential to alleviate α-syn pathology in multiple in vitro and in vivo α-synucleinopathic models. Regulation of neuronal α-syn proteostasis underlies the therapeutic function of astrocytes. Specifically, VM-derived astrocytes inhibited neuronal α-syn aggregation and transmission in a paracrine manner by correcting not only intraneuronal oxidative and mitochondrial stresses but also extracellular inflammatory environments, in which α-syn proteins are prone to pathologic misfolding. The astrocyte-derived paracrine factors also promoted disassembly of extracellular α-syn aggregates. In addition to the aggregated form of α-syn, VM astrocytes reduced total α-syn protein loads both by actively scavenging extracellular α-syn fibrils and by a paracrine stimulation of neuronal autophagic clearance of α-syn. Transplantation of VM astrocytes into the midbrain of PD model mice alleviated α-syn pathology and protected the midbrain dopamine neurons from neurodegeneration. We further showed that cografting of VM astrocytes could be exploited in stem cell–based therapy for PD, in which host-to-graft transmission of α-syn pathology remains a critical concern for long-term cell therapeutic effects.

## Significance

Intraneuronal inclusions of misfolded α-synuclein (α-syn) and prion-like spread of the pathologic α-syn contribute to progressive neuronal death in Parkinson’s disease (PD). Despite the pathologic significance, no efficient therapeutic intervention targeting α-synucleinopathy has been developed. In this study, we showed that astrocytes, especially those cultured from the ventral midbrain, substantially alleviate neuronal α-syn pathology by regulating a series of the proteostasis procedures associated with formation, transmission, disaggregation, and clearance of toxic α-syn aggregates in multiple in vitro and in vivo α-synucleinopathic models. Based on these findings, the therapeutic actions of astrocytes are proposed for use in relieving α-syn–mediated neuronal toxicity and in setting up a desirable cell-based therapy free from host-to-graft α-syn propagation in PD.

Parkinson’s disease (PD) is a prevalent neurodegenerative disorder with movement symptoms characterized by progressive loss of dopaminergic (DA) neurons in the substantia nigra (SN) pars compacta of the midbrain with the concomitant loss of nigrostriatal DA neurotransmission. A pathologic hallmark of PD is intraneuronal inclusion of α-synuclein (α-syn) aggregates, called Lewy bodies and Lewy neurites. The α-syn aggregates cause various cellular dysfunctions including mitochondrial impairment, defective endoplasmic reticulum (ER) function, autolysosomal pathways, and synaptic and nuclear dysfunctions (1, 2). Aggregated α-syn is released from neuronal cells and acts as a ligand for patterned recognition receptors, which activate inflammatory responses in glial cells (3, 4). Furthermore, the pathologic protein aggregates undergo neuron-to-neuron transmission in a prion-like fashion (reviewed in ref. 5). The α-syn propagation and neuroinflammation are closely related to disease progression and clinical severity (6).

Given its pathologic significance, the α-syn proteinopathy is a major research focus to develop disease-modifying therapies for PD and other synucleinopathic disorders such as Lewy body dementia, multiple system atrophy, and certain forms of Alzheimer’s disease. However, no therapeutic intervention to effectively eliminate the pathologic α-syn has been developed to date. In addition to the diseased conditions, the aggregated species of α-syn are also accumulated in the midbrain SN during normal aging, but not in young brain tissues (7), suggesting the existence of homeostatic regulation to prevent and resolve α-syn aggregation in young and healthy brains. This suggests homeostatic functions may be useful in developing therapeutic tools. In this regard, astrocytes are a prime cell type to be studied for therapeutic applications, as this glia cell type has multiple functions related to maintaining brain homeostasis, including those for correct functioning of neurons and protecting neuronal cells from pathologic insults (reviewed in ref. 8). Recent studies have shown the capacity of astrocytes to efficiently take up and degrade α-syn (9–12). Due to the astrocyte scavenging effect, α-syn inclusions are usually not detected in astrocytes of PD patients except in advanced stages of the disease (13–18). In addition, in contrast to efficient transmission of neuronal α-syn proteins into astrocytes, α-syn transfer from astrocytes to neuronal cells is inefficient (11), collectively suggesting a role for astrocytes in scavenging α-syn rather than in spreading it. The role of homeostatic astrocytes in α-syn pathology, however, remains to be unraveled.

In this study, we showed that astrocytes, especially those cultured from the ventral midbrain (VM), the brain region primarily affected in PD, substantially alleviate neuronal α-syn pathology by regulating a series of the proteostasis procedures associated with formation, transmission, disaggregation, and clearance of toxic α-syn aggregates. Upon transplantation, VM-type astrocytes efficiently eliminated pathologic α-syn accumulation and α-syn–induced DA neuron degeneration in the midbrain of PD model mice. We further show that host-to-graft propagation of toxic α-syn, reported as a critical concern in the cell-based therapeutic approach for PD (19, 20), was greatly prevented by cografting the cultured astrocytes. Based on these findings, the therapeutic actions of astrocytes are proposed for use in relieving α-syn–mediated neuronal toxicity and in setting up a desirable cell-based therapy free from host-to-graft α-syn propagation in PD.

## Results

### Astrocytes Cultured from VM Reduce the Aggregated and Monomer Forms of α-Syn in VM-Derived Midbrain-Type Dopamine (mDA) Neuron-Enriched Cultures.

The aim of this study was to assess whether astrocytes could be utilized to treat α-syn pathology in the context of PD. Based on the inherent heterogeneity of astrocytes derived from brain regions (21–25), we cultured astrocytes from two rodent brain regions that included the classical nondopaminergic brain region cortex (Ctx) and dopaminergic VM, which is primarily affected in PD (schematized in *SI Appendix*, Fig. S1*A*). As a control, neural stem/precursor cells (NSCs) were cultured from Ctx of rodent embryonic brains. The majority of cells (70 to 85%) in the Ctx and VM astroglial cultures commonly expressed astrocyte-specific markers (GFAP, GLAST, CD44, GLT-1, S100β, ALDH1L1, and Aqp4) (*SI Appendix*, Fig. S1 *B* and *C*, *Left*), and the regional identities of the cultures were faithfully maintained by expressing their brain-region-specific marker expressions (*SI Appendix*, Fig. S1 *C*, *Right*). None and a few (<1%) were positive for O4 (oligodendrocyte) and Iba1 (microglia), respectively.

Formation of α-syn aggregates in PD patients and experimental models has shown to be induced by increased expression of α-syn (26–28). To set up an in vitro cellular model for α-synucleinopathic PD in which misfolded α-syn oligomers and fibrils are formed and propagated among neuronal cells, NSCs derived from the VM of rodent embryos (mouse at embryonic day [E]10.5 or rat at E12) were transduced with lentiviruses expressing human α-syn (tagged with green fluorescent protein [GFP]). They were then differentiated (schematized in *SI Appendix*, Fig. S1*D*). The NSCs derived from early embryonic VM preferentially differentiated into neuronal cells (29), and 20 to 30% of TuJ1+ differentiated neurons were mDA neurons expressing proteins specific to DA homeostasis (TH and AADC) and midbrain-type DA neurons (Nurr1, Foxa2, and Lmx1a) (*SI Appendix*, Fig. S1*E* and ref. 30). The proportion of DA neurons expressing midbrain-specific markers reflected the adult mouse VM (percent TH+ DA neurons of total NeuN+ neurons in the mouse VM at 23 wk of age: 23.8%). Exogenous α-syn expressions were widely detected in the mDA neurons (*SI Appendix*, Fig. S1*E*). To facilitate α-syn aggregate formation and propagation in the cultured mDA neuronal cells, the differentiated cultures were treated with human α-syn preformed fibrils (α-syn-PFFs) (*SI Appendix*, Figs. S1*D* and S2*A*), which act as a seed for intracellular α-syn aggregation in neuronal cells (31, 32). In this combined α-syn treatment condition, α-syn aggregation was abundantly detected 14 to 20 d after PFF treatment, in the estimation by staining with Thioflavin S dye visualizing misfolded protein aggregates, as well as by immunocytochemical analysis against phosphorylated α-syn at serine 129 (p129-α-syn), pathologically modified forms of α-syn associated with toxic α-syn fibril formation (33) (*SI Appendix*, Fig. S2*B*). Western blot (WB) analysis further showed a great increase in oligomer forms of α-syn both in the Triton X-soluble and insoluble (sodium dodecyl sulfate [SDS]-soluble) fractions prepared from the mDA neuronal cultures treated with the exogenous α-syn (*SI Appendix*, Fig. S2*C*). Collectively, these results indicate that we established an appropriate in vitro platform to evaluate the effect of therapeutic candidates on α-syn aggregation in the disease context of PD.

**Fig. 1.**
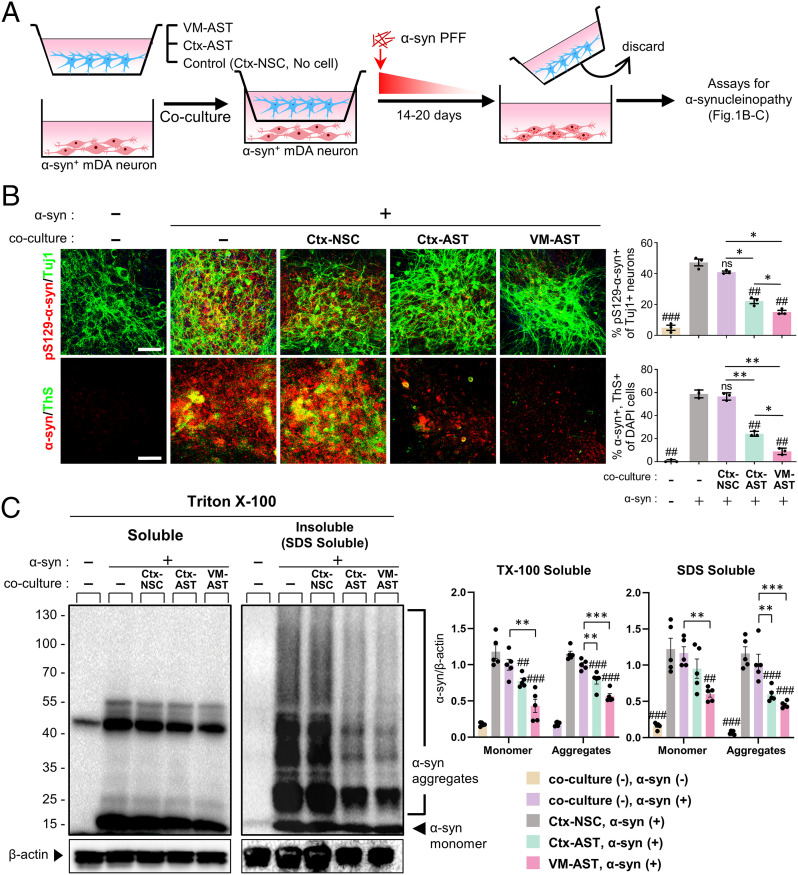
Cultured astrocytes reduce the aggregated and monomer forms of α-syn protein in the in vitro α-synucleinopathic PD cellular model. (*A*) Schematics of the coculture experimental procedures. Astrocytes derived from the VM or Ctx of rat pups (undifferentiated Ctx-NSCs as a control; *SI Appendix*, Fig. S1 *A*–*C*) were cocultured with α-syn–overexpressing mDA neurons (*SI Appendix*, Fig. S1 *D* and *E*) using the culture insert (1 x10^5^ cells plated each in the upper and lower chambers, separated using a polycarbonate membrane with 0.4-µm pore size). As another control, the culture insert without cell seeding was used. Media containing sonicated α-syn PFF (1 µg/mL) was added to both the upper and lower compartments and followed by gradual dilution of α-syn PFF by changing half the medium every other day for 14 to 20 d. (*B*) α-syn aggregation detected by immunoreactivity against pS129-αsyn and Thioflavin S staining. (*C*) WB-based detection of monomer and aggregate forms of α-syn in the Triton X-100–soluble and –insoluble (SDS soluble) fractions. Significant differences from the α-syn-treated (+) and without cocultured cells (−) control at ^##^*P* < 0.01 and ^###^*P* < 0.001 and between the groups indicated at *P < 0.05, **P < 0.01, and ***P < 0.001. *n* = 3 (*B*) and 5 (*C*), one-way ANOVA, followed by Bonferroni post hoc analysis. ns, no significance. (Scale bars: 25 μm.)

To assess astrocyte functions on neuronal α-synucleinopathy, the α-syn–overexpressing mDA neuronal cells (7 d after differentiation) were cocultured with Ctx, VM astrocytes, or Ctx NSCs (as control) using a culture insert device (Fig. 1*A*). Of note, the number of neuronal cells positive for Thioflavin S/pS129-α-syn as compared to the control cultures cocultured with Ctx NSC or those without coculture (no cells in the insert) were greatly reduced in the cultures cocultured with astrocytes, and greater reduction was observed by coculturing with VM astrocytes (Fig. 1*B*). The astrocyte-coculturing effect on α-syn aggregate formation was also manifested in multiple consecutive WB analyses exhibiting significantly lower levels of α-syn aggregates in the cultures cocultured with VM or Ctx astrocytes as compared to the control cultures (Fig. 1*C*). In addition to the aggregate levels, the monomer form of α-syn was also reduced in the cocultures with astrocytes, resulting in a decrease of total α-syn levels by coculturing with astrocytes and a more significant decrease with VM astrocytes. The α-syn clearance effects were further manifested using another type of coculturing, in which α-syn+ mDA neurons were directly mixed with VM or Ctx astrocytes (or Ctx NSCs as a control) (*SI Appendix*, Fig. S3). These findings together indicate that astrocytes, especially those derived from VM, can address α-synucleinopathy by reducing α-syn protein level as well as by inhibiting α-syn misfolding (and/or activating α-syn disassembly).

### Astrocytes Inhibit Intraneuronal α-Syn Aggregation and Neuron-to-Neuron Transmission in a Paracrine Manner.

**Fig. 2.**
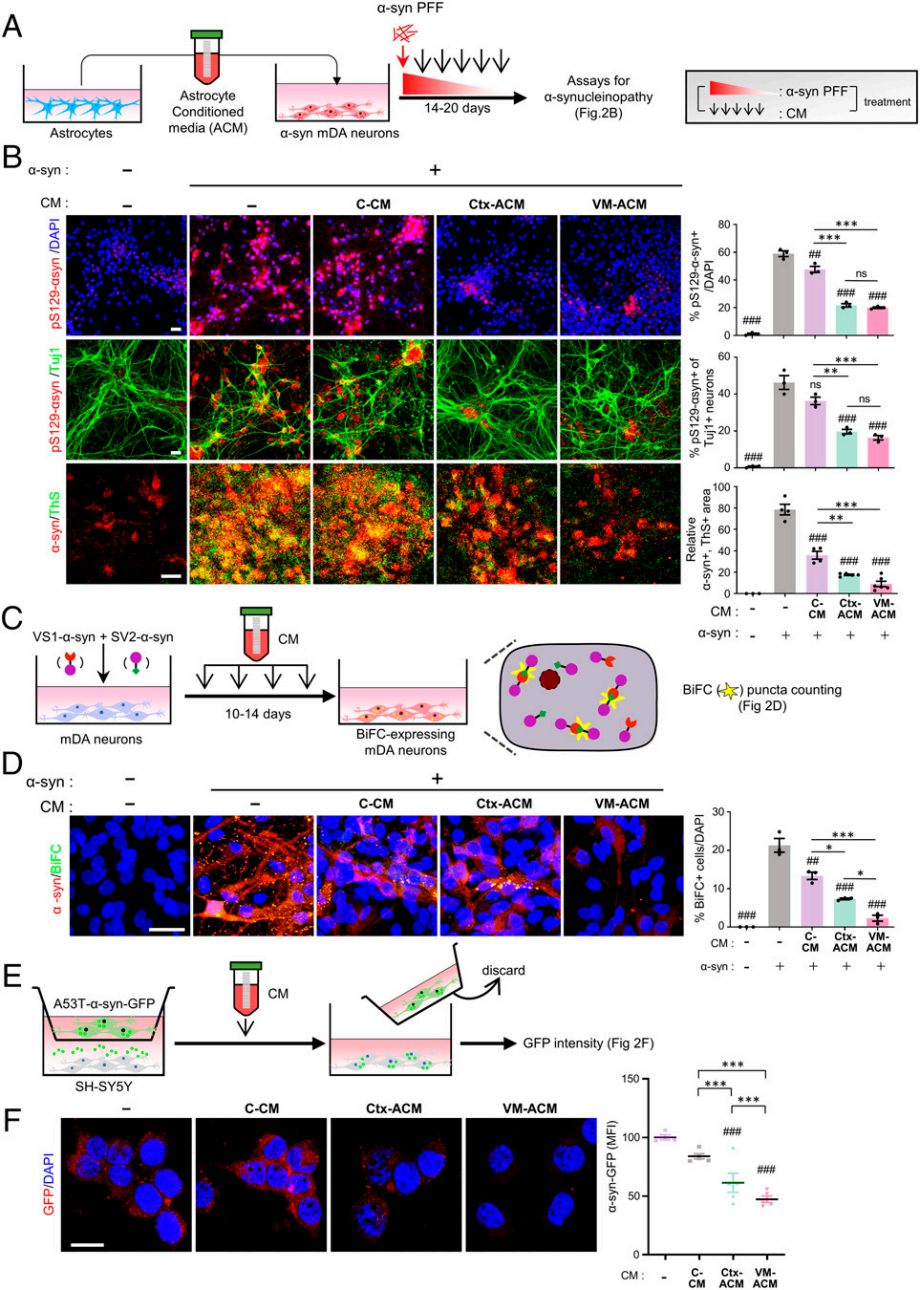
Paracrine factors released from cultured astrocytes prevent intraneuronal oligomerization and interneuronal propagation of α-syn. (*A* and *B*) Paracrine effect of cultured astrocytes on the levels of α-syn aggregation. Media conditioned in astrocyte (ACM) or control Ctx-NSC (C-CM) cultures were collected and treated to α-syn–expressing mDA neuron-enriched cultures. Fourteen to 20 d after α-syn PFF treatment, α-syn aggregation was detected by immunoreactivity against pS129-αsyn and Thioflavin S staining. (*C* and *D*) Intracellular di-/oligomerization detected by α-syn BiFC system. (*C*) Schematic of the experimental procedures with BiFC system. BiFC+ inclusions (yellow puncta) are counted in *D*. (*E* and *F*) Interneuronal α-syn transmission. In the dual-chamber device, in which SH-SY5Y cells transduced with GFP-labeled A53T α-syn and those of nontransduced were plated in the upper and lower chambers, respectively (1 × 10^4^ cells in each chamber, schematized in *E*), neuron-to-neuron α-syn transmission was estimated by counting A53T-αsyn-GFP puncta intensity (*F*). GFP fluorescence intensity was measured using ImageJ software and presented as mean fluorescence intensity (MFI). Significant differences from the α-syn–treated (+) and CM-untreated (−) control at ^##^*P* < 0.01 and ^###^*P* < 0.001 and between the groups indicated at *P < 0.05, **P < 0.01, and ***P < 0.001, *n* = 3 to 5 cultures (*B*–*D*), 13 to 89 cells (*F*), one-way ANOVA. ns, no significance. (Scale bars: 25 μm.)

We supposed that the observed astrocytic effects were obtained by 1) molecules released from the astrocytes and/or 2) cell-autonomous astrocyte functions to scavenge extracellular α-syn by endo-/phagocytic uptake and degradation. To assess if factors secreted from the cultured astrocytes are involved in the observed astrocyte-mediated reduction of α-syn aggregates, a medium was conditioned in the VM, Ctx astrocyte cultures (or Ctx NSC cultures as the control), and the conditioned medium (CM) was added to the α-syn–transduced mDA neuronal cultures supplemented with α-syn-PFF (Fig. 2*A*). Compared with the control CM (C-CM)-treated (or CM-untreated control cultures), the percent pS129-α-syn+ out of total TuJ1+ neurons and pS129-α-syn+/Thioflavin+ cells was significantly reduced in the cultures treated with astrocytic CM (ACM) (Fig. 2*B*). The ACM-mediated effect was also observed in α-syn WB analysis (*SI Appendix*, Fig. S4*A*).

We further scrutinized the astrocytic paracrine functions by determining their roles in a series of α-syn proteostasis processes. First, we asked if the observed ACM-mediated reduction of the misfolded α-syn aggregation was obtained by regulating intraneuronal α-syn oligomer formation. To this end, we utilized a bimolecular fluorescence complementation (BiFC) system (34) in which yellow fluorescence is achieved by dimerization or oligomerization between α-syn fused to the amino (N) terminus (V1S) and that fused to the α-syn carboxy (C) terminus (SV2) fragment of Venus, a variant of yellow fluorescence protein (YFP) (Fig. 2*C*). Primary cultured VM NSCs were transduced with a mixture of the V1S and SV2 constructs, which were differentiated into neurons, and the potential of intracellular α-syn dimer-/oligomerization was estimated by counting BiFC-YFP puncta. Neither the transduction with V1S nor SV2 alone produced BiFC fluorescence. The BiFC puncta were readily formed in the neuronal cells expressing both the BiFC-α-syn constructs. As anticipated, the numbers of BiFC puncta were greatly reduced in the cultures treated with ACM as compared to that with C-CM and CM-untreated, and the inhibitory effects by VM-ACM were significantly greater than those of Ctx-ACM (Fig. 2*D*). The ACM effect was further tested in SH-SY5Y neuronal cells expressing GFP-fused A53T α-syn (A53T α-syn-GFP), a pathogenic mutant prone to β-sheet structured aggregation (35), by counting cytoplasmic α-syn-GFP puncta, which can be used to quantify α-syn aggregation (36, 37) (*SI Appendix*, Fig. S4*B*). The treatment of ACM, especially that derived from VM astrocytes, dramatically lowered the GFP puncta numbers (*SI Appendix*, Fig. S4*C*), confirming paracrine function of cultured astrocytes to inhibit intraneuronal α-syn oligomerization.

Next, we estimated the ACM effect on cell-to-cell α-syn transmission by adopting a dual-chamber system, in which SH-SY5Y neuronal cells expressing GFP-labeled A53T α-syn were placed in the upper chamber and nontransduced neurons were in the lower chamber (38, 39) (Fig. 2*E*). Sixteen hours after coculturing, the interneuronal α-syn transmission (as estimated by GFP fluorescence intensity in the recipient cells of the lower chamber) significantly decreased in the presence of ACM, and it was lowest in the cultures treated with the ACM derived from VM astrocytes (Fig. 2*F*).

Finally, to assess the combined α-syn transmission + aggregation efficiency, we modified the BiFC system by transducing neuronal cultures individually with the V1S and SV2 constructs, instead of transduction with the constructs together (*SI Appendix*, Fig. S4*D*). Neither the neuronal cells expressing V1S nor those expressing SV2 produced BiFC fluorescence. BiFC fluorescence only appeared by coculturing the V1S- and SV2-expressing neuronal cells, indicating the α-syn fragments were readily secreted, transferred, and oligomerized in neighboring cells. As shown in *SI Appendix*, Fig. S4*E*, BiFC puncta greatly decreased in the cocultures treated with ACM. Similarly, the ACM treatment significantly decreased double-labeled (yellow) puncta in SH-SY5Y neuronal cells expressing A53T α-syn-GFP cocultured with those expressing A53T α-syn-mCherry (*SI Appendix*, Fig. S4 *F* and *G*). Collectively, these findings suggest that astrocytes, especially VM-derived astrocytes, inhibit interneuronal α-syn transmission as well as intraneuronal α-syn aggregate formation in a paracrine manner.

### Molecules Released from Astrocytes Correct Intra- and Extracellular Environments Facilitating α-Syn Aggregate Formation.

Oxidative and mitochondrial stress is the prime cause of pathologic intraneuronal α-syn aggregation (40–42). Mitochondrial damage causes increased reactive oxygen species (ROS) with defective mitochondrial antioxidant functions as well as increased Ca^2+^/calpain-mediated cleavage of α-syn with defected mitochondrial Ca^2+^ buffering capacity, ultimately resulting in oxidized/nitrosyl and truncated α-syn, respectively, which are prone to undergo pathologic aggregation (reviewed in ref. 43). In addition, interneuronal α-syn transmission is also sensitive to folding states and is promoted under oxidative stress conditions (44, 45). In accordance with acknowledged neurotrophic actions by the paracrine factors released from astrocytes, intraneuronal oxidative and mitochondrial stresses, estimated by DCF staining and Mito-Sox, were greatly reduced in the mDA neuronal cultures treated with the VM-ACM and Ctx-ACM (*SI Appendix*, Fig. S5 *A* and *B*).

Toxic α-syn aggregation and propagation are also promoted in extracellular inflammatory environments, mainly by reactive microglia, the resident brain immune cells (reviewed in ref. 46). To test the effect of cultured astrocytes for modulating brain inflammation, we cultured microglia from mouse brain tissue. ACM treatment reduced proinflammatory cytokine expression in the microglia (activated by LPS+ATP) (*SI Appendix*, Fig. S6*A*). Recent studies have shown a strong association of microglial NOD-like receptor family pyrin domain-containing 3 (NLRP3) inflammasome activation with PD protein pathology (47, 48). Secreted levels of cleaved caspase-1 and interleukin (IL)-1β, the products of activated NLRP3 inflammasomes, were markedly decreased in microglia cultures treated with ACM and more dramatically by VM-ACM (*SI Appendix*, Fig. S6*B*). Consistent to the previous studies showing antiinflammatory functions of NSCs (49–51), C-CM prepared from Ctx-NSCs also reduced the microglial proinflammatory cytokine expression and NLRP3-inflammasome activation (*SI Appendix*, Fig. S6). These findings suggest that astrocytes exert anti–α-synuleinopathic functions partly by correcting intra- and extraneuronal toxic/inflammatory environments.

### Astrocytes Promote Neuronal Autolysosomal Clearance of α-Syn in a Non-Cell-Autonomous Manner.

It is noted that in addition to the reduced α-syn aggregation, total levels of neuronal α-syn proteins were also lowered by the astrocyte coculture (Fig. 1*C*) or by the ACM treatment (*SI Appendix*, Fig. S4*A*). Dysregulation of autophagy in diseased neurons is a main cause of toxic α-syn accumulation (52). The neuronal autophagic clearance of α-syn is regulated by paracrine factors (53) and potentially by those released from astrocytes (54–56). Based on these findings, we asked if soluble factors released from astrocytes could facilitate neuronal autophagic clearance of α-syn, and thus might ultimately contribute to the observed astrocyte-mediated decrease of α-syn protein. In the basal condition, we found an increase of the autophagosome component LC3II protein levels with a decrease of p62 in the ACM-treated mDA neuronal cultures as compared to the C-CM–treated or CM-untreated cultures (*SI Appendix*, Fig. S7 *A* and *B*). The p62 protein levels increased in the presence of Bafilomycin A1, an inhibitor of autolysosomal degradation at the late stage of autophagy, and those in the ACM-treated cultures became indistinguishable from the control levels in the Bafilomycin A1-treated condition, indicating that the decrease of the basal p62 levels in the ACM-treated cultures is due to increased autolysosomal clearance. EBSS treatment, a nutrient starvation condition to promote initial autophagosome formation, caused an increase of LC3II in both ACM-treated and control cultures. The ACM treatment-induced increase in LC3II levels observed in the basal condition was maintained in the EBSS-treated condition, collectively suggesting an ACM effect to increase autophagic flux in neuronal cells. We further examined the neuronal autophagic flux using the mCherry-GFP-LC3 tandem fluorescent probe, which emanates both mCherry and GFP signals in the autophagosome stage but only mCherry fluorescence in the stage of autolysosome due to the acidic pH of the autolysosome (*SI Appendix*, Fig. S7*C*). The ACM-treated cultures showed increases in both the autophagosome (yellow puncta) and autolysosome (red puncta) numbers, confirming the increase of autophagy flux in the ACM-treated cultures (*SI Appendix*, Fig. S7 *D*–*F*). To further validate that the reduced α-syn proteins are mediated by the increase in autophagic clearance, we checked the colocalization of pS129-α-syn with the autophagosome (yellow puncta) and autolysosome (red puncta). Along with the ACM effect to reduce pS129-α-syn+ cells (Fig. 2*B*), confocal microscopic examination on the single cellular level showed that pS129-α-syn+ puncta/cell were also greatly lowered in the ACM-treated neuronal cells (*SI Appendix*, Fig. S7 *G* and *H*). Of note, the decrease in the pathologic α-syn puncta concurred with a significant increase of pS129-α-syn colocalization with the autolysosome puncta (pink puncta, indicated by white arrows in *SI Appendix*, Fig. S7 *G* and *I*). These findings together suggest that neuronal autophagy flux and thus autolysosomal clearance of α-syn are promoted by soluble factors released from astrocytes.

### Extracellular α-Syn Aggregates Are Dissolved by the Molecules Released from Astrocytes.

**Fig. 3.**
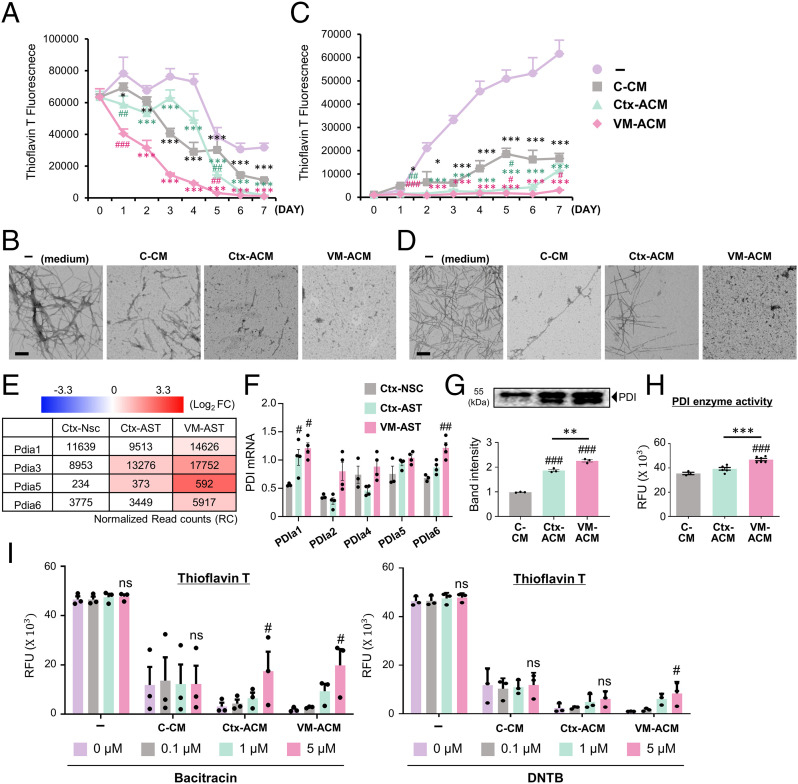
Extracellular α-syn disassembling and antiaggregation activities of astrocyte-derived paracrine molecules. (*A* and *C*) Time-course Thioflavin T assays to monitor disassembly of α-syn fibrils (*A*) and aggregate formation from α-syn monomers (*C*). Alpha-synuclein-PFFs (*A*) and monomers (*C*) were incubated at 37 °C with the VM-ACM, Ctx-ACM, C-CM, or medium (as a control). Thioflavin T assays were done at the time points of incubation indicated. (*B* and *D*) TEM images for the dissembled or aggregated α-syn at 7 (*A* and *C*) days after incubation. (Scale bars: 200 nm.) Significantly decreased from no CM (−) at *P < 0.05, **P < 0.01, and ***P < 0.001 and C-CM at ^#^*P* < 0.05, ^##^*P* < 0.01, and ^###^*P* < 0.001, *n* = 3 independent experiments, one-way ANOVA. (*E*–*H*) PDI mediates the ACM-mediated α-syn disassembly. (*E* and *F*) Messenger RNA expression of PDI isozymes detected by RNA-seq (accession no. GSE106216) (*E*) and real time-PCR (*F*) analyses. Expression in RNA-seq data is represented by the read count (RC) normalized using DESeq2 (v1.32) with the relative log expression (RLE) method (inside box) and Log2[Astrocyte/control Ctx-NSC] (color intensities). Significantly different from Ctx-NSC at ^#^*P* < 0.05 and ^##^*P* < 0.01. (*G* and *H*) Secreted PDI protein levels and enzyme activities determined in the CMs. RFU, relative fluorescence units. Significantly different from C-CM at ^###^*P* < 0.001 and between the groups indicated at **P < 0.01 and ***P < 0.001, *n* = 3 to 6 of three independent experiments. (*I*) ACM-mediated α-syn disassembly activity blunted by the treatment of PDI inhibitors. α-syn PFFs were incubated with VM (Ctx)-ACM (or C-CM) in the presence or absence of the PDI inhibitors bacitracin and DTNB. Five days after incubation, α-syn disaggregation levels were determined by Thioflavin T assays. Significant differences from the inhibitor-untreated (0 μM) at ^#^*P* < 0.05, *n* = 3 independent experiments, one-way ANOVA. ns, no significance.

Toxic α-syn aggregates from donor neuronal cells are released to extracellular space through nonspecific means including cell death or through specific, tightly regulated cellular pathways such as exocytosis (57). The extracellular α-syn fibrils act as a damage-associated molecular pattern to modulate the immune response in the central nervous system (CNS) (58) as well as a source for interneuronal propagation of the α-syn pathology (59). Thus, extracellular α-syn is also suggested as a therapeutic target (60). The time-course Thioflavin T assay in the cell-free condition demonstrated that α-syn fibrils were drastically dissembled in the presence of ACM when α-syn-PFF (50 μg) was incubated in test tubes in the absence or presence of ACM (or C-CM) (Fig. 3*A*). The ACM-induced disaggregation of α-syn fibrils was further confirmed by transmission electron microscopy (TEM) analysis (Fig. 3*B*).

Pathologic α-syn aggregation is generally considered to occur in intracellular environments. However, a portion of α-syn monomers is secreted (61, 62), and extracellular α-syn is more prone to aggregation than the cytosolic protein (62). Thus, we also tested the potential role of astrocyte-derived soluble factors in regulating α-syn aggregate formation in the cell-free condition. α-syn monomer (50 μg) incubated in a test tube at 37 °C gradually aggregated over 7 d based on the Thioflavin T assay and TEM examination (Fig. 3 *C* and *D*). Of note, α-syn fibril formation was dramatically prevented in the presence of ACM. VM-ACM showed greater effects to promote the α-syn dissociation (Fig. 3 *A* and *B*) and to inhibit fibril formation (Fig. 3 *C* and *D*) compared to Ctx-ACM. The C-CM derived from the control Ctx-NSCs also exhibited dissociation and antifibril formation effects, but these were much lower than those of ACMs. Collectively, these findings suggest that astrocytes inhibit extracellular deposits of α-syn aggregates in a paracrine manner by preventing α-syn fibrillization as well as by promoting disassembly of α-syn fibrils.

Protein disulfide isomerase (PDI), a chaperone and oxidoreductase, is one of the few molecules known for dissolving or preventing the aggregation of misfolded proteins (63). This enzyme, mainly located in the ER, is released and also acts at extracellular locations (64). In our RNA-sequencing (RNA-seq) data (Gene Expression Omnibus accession no. GSE106216), the expression of multiple PDI isoenzymes in cultured astrocytes was greater than those of the control Ctx-NSCs (Fig. 3*E*), and the up-regulated pattern of the PDIs in the astrocytic cultures was confirmed using real-time PCR (Fig. 3*F*). In accordance with a secretory property, PDI protein and enzyme activity were readily detected in all the CMs used, but those in the ACMs were greater than in the C-CM and greatest in the VM-ACM (Fig. 3 *G* and *H*). The ACM-mediated α-syn disaggregation was largely abolished in the presence of the PDI inhibitors (bacitracin and DTNB) (Fig. 3*I*), collectively indicating an involvement of PDI in the observed ACM activity to dissolve α-syn aggregates.

In the RNA-seq data (GSE106216), the expression of the proteins with the reported enzyme activities to degrade extracellular α-syn protein (ECE1, ECE2, neurosin, and MMP2) (65, 66) was greater in the Ctx and VM astrocyte cultures than in the control Ctx-NSC cultures (*SI Appendix*, Fig. S8*A*). Thus, we tested if astrocyte-derived molecules can directly degrade α-syn protein and thus contribute to extracellular α-syn clearance. To this end, the purified monomer form of α-syn protein was incubated in the presence or absence of ACM (or C-CM), and the α-syn protein level change was monitored for 7 d of incubation. No significant time-course changes in α-syn protein levels were detected in the CM-treated reactions (*SI Appendix*, Fig. S8*B*).

### Astrocytic Phagocytosis and Clearance of α-Syn.

**Fig. 4.**
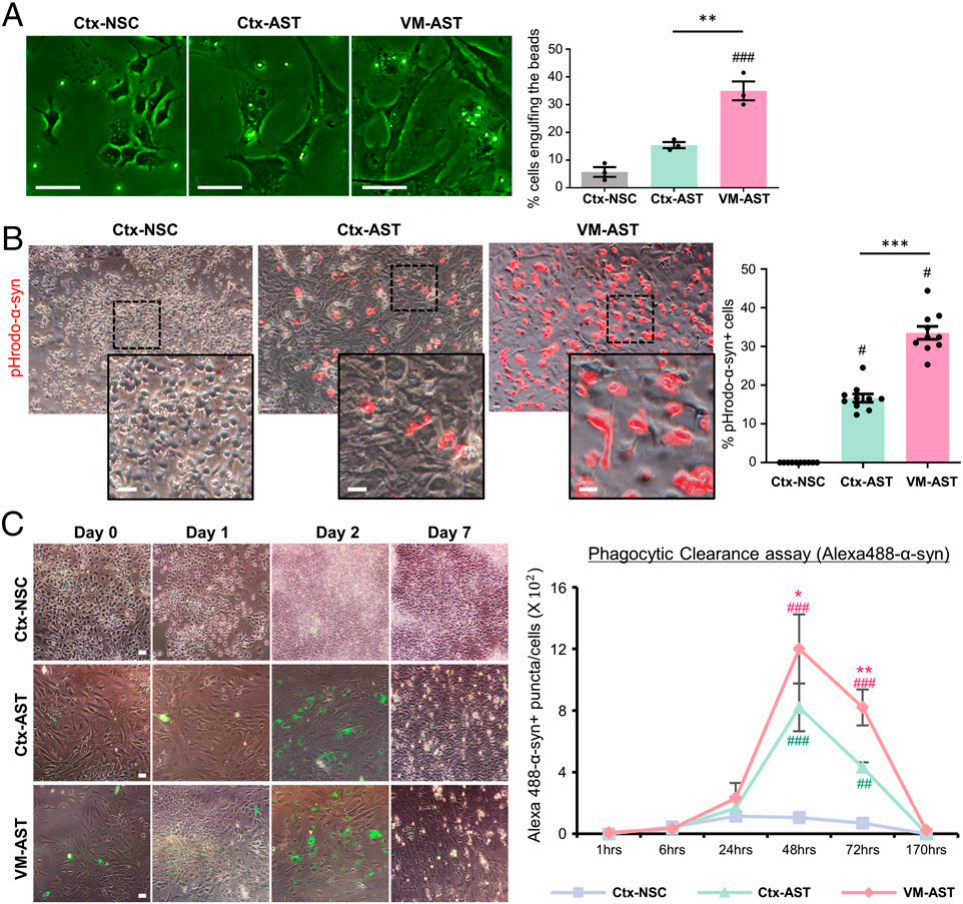
Phagocytic clearance of α-syn by cultured astrocytes. (*A*) General phagocytic activity determined using fluorescent latex beads (F-13081). Cultured astrocytes (Ctx-NSCs as control) were incubated with the latex beads for 24 h and washed followed by quantifying percent cells engulfing the beads. (*B*) Activity to phagocytose α-syn determined by incubating with α-syn PFF labeled with pHrodo, a pH-sensitive fluorescent dye. (*C*) Phagocytic clearance of α-syn. Cultured astrocytes were incubated with Alexa 488-labeled α-syn-PFF for 24 h. Percent cells containing Alexa 488-α-syn was chased for 7 d after withdrawal of the fluorescence-labeled α-syn-PFF. Significant differences from Ctx-NSC at ^#^*P* < 0.05, ^##^*P* < 0.01, and ^###^*P* < 0.001 and from Ctx-Ast at *P < 0.05, **P < 0.01, and ***P < 0.001, *n* = 3 to 10, one-way ANOVA. (Scale bars: 25 μm.)

The uptake and clearance of misfolded/aggregated proteins is a key process to control extracellular deposition of α-syn aggregates and the spreading and progression of the disease (reviewed in ref. 67). Thus, the final question to be addressed is whether astrocytes facilitate the removal of toxic α-syn by phagocytic clearance. General phagocytic activity was assessed by incubating cells with fluorescent latex beads (F-13081; Thermo Fisher Scientific) for 24 h and quantifying the pecent cells engulfing the beads. In addition, to assess α-syn–specific phagocytosis, cells were incubated with α-syn PFF labeled with pHrodo, a pH-sensitive fluorescent dye, which allowed us to quantify α-syn taken up into the acidic milieu of the phagolysosomes. Twenty-four hours after incubation, beads were engulfed in a portion of the control Ctx-NSCs, while pHrodo-α-syn was scarcely detected in the control cultures (Fig. 4 *A* and *B*). In identical conditions, cultured astrocytes showed potent general and α-syn–specific phagocytic activities. It was further noted that the phagocytic activities of VM astrocytes were significantly greater than those of Ctx astrocytes. When astrocytes were incubated with Alexa 488-labeled α-syn for 24 h, internalized Alexa 488-α-syn reached its peak 1 d after withdrawal of the α-syn, and it was almost completely cleared during the next 5 d (Fig. 4*C*). These findings are consistent with recent literature showing that α-syn toxic species can be efficiently internalized by astrocytic cells and degraded through the lysosomal pathway (9–11). This study further suggests that astrocytes from VM have stronger activity for scavenging α-syn than those from cortices. Multiple studies have shown that the cell-surface proteins heparan sulfate proteoglycan (HSPG) and low-density lipoprotein receptor-related protein 1 (LRP1) mediate uptake and degradation of Tau and α-syn associated with Alzheimer’s disease and PD (68–73). In the RNA-seq data (accession no. GSE106216), VM and Ctx astrocytes expressed greater levels of HSPG (11- and 10-fold) and LRP1 (2.5 and 2.5 times, respectively) than the control NSCs. Although our data showed that astrocytes efficiently clear up the phagocytosed α-syn, a small amount of uncleared α-syn can act as a seed to induce pathologic aggregates in astrocytes, like in neuronal cells. As expected, incubation of SH-SY5Y neuronal cells with α-syn PFF for 24 h sufficiently induced the formation of α-syn oligomers in WB analysis, which increased gradually during 3 to 12 d after withdrawal of exogenous α-syn PFF (*SI Appendix*, Fig. S9*A*). By contrast, the aggregate bands were only detected in the early days but were reduced or disappeared during the rest of the postwithdrawal period in the astrocyte cultures (*SI Appendix*, Fig. S9 *B* and *C*). Collectively, these findings suggest that astrocytes efficiently uptake and degrade misfolded α-syn without causing intracellular α-syn aggregation/propagation by the internalized exogenous α-syn.

### Therapeutic Potential of Grafted VM Astrocytes in an α-Syn–Induced PD Animal Model.

**Fig. 5.**
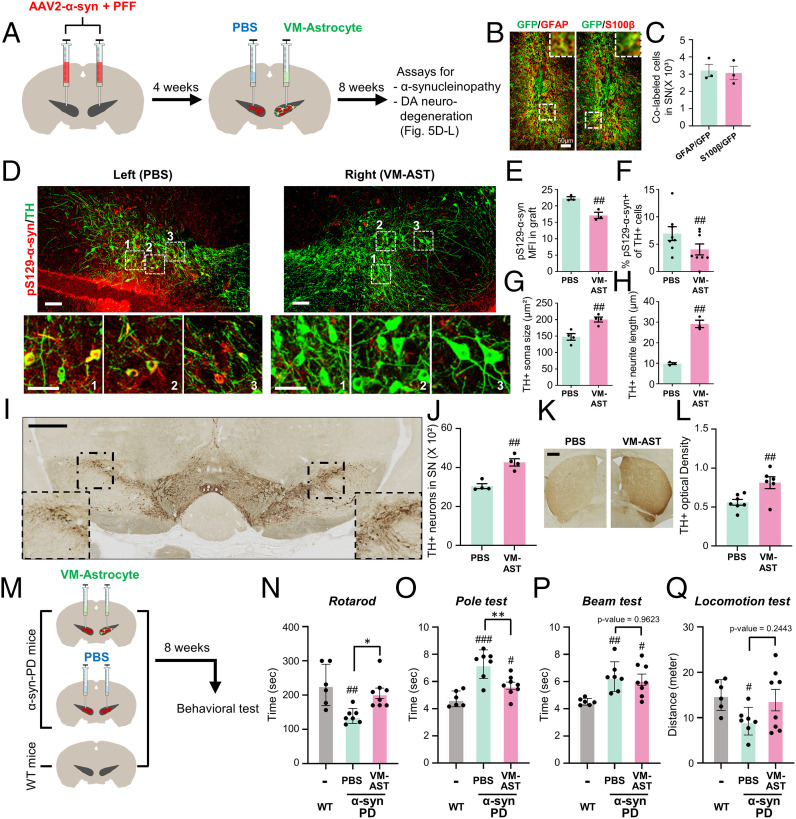
In vivo therapeutic functions of cultured astrocytes in α-synucleinopathic PD model mice. (*A*) Schematic of the experimental procedure. α-synucleinopathic PD (α-syn-PD) model mice were generated by bilateral injection of α-syn–expressing AAV2+α-syn PFF into the midbrain SN. Cultured VM astrocytes were transplanted into the right side of the midbrain SN in the PD mice (PBS injection [sham-operated] to the left SN). Two months posttransplantation, α-syn pathology and DA neuron degeneration in the right SNs transplanted with the astrocytes were compared with those in the left SNs (sham-operated) of the same animals. (*B* and *C*) Engraftment of transplanted astrocytes. α-syn-PD mice were grafted with VM astrocytes transduced with GFP-expressing lentivirus. Two months later, efficiency of cell engraftment was assessed by counting GFP+ cells coexpressing the astrocyte-specific markers GFAP and S100β. (*Insets*) Enlarged images of boxed areas. (*D*–*F*) Alpha-synucleinopathy assessed by percent pS129-αsyn immune intensities and pS129-αsyn+/TH+ cells. Boxed areas are enlarged in the lower panel. (*G* and *H*) DA neuron degeneration assessed by soma sizes (*G*) and fiber lengths (*H*) of the TH+ DA neurons (immunefluorescence stained), numbers of DA neurons (*I* and *J*), and TH+ fiber intensity in the striatum (*K* and *L*) (DAB-stained). (*Insets*) Enlarged images of boxed areas. Significant differences from the left side of SN of the identical animals at ^##^*P* < 0.01 and ^###^*P* < 0.001. *n* = 3 to 8 mice, paired *t* test. (*M*–*Q*) Behavioral assays. Schematic of the experimental procedure (*M*). VM astrocytes were grafted bilaterally into the SNs of α-syn-PD mice, and behaviors of the mice were assessed compared with sham-operated (PBS-injected) and age-matched WT mice. Behaviors of the PD mice were assessed by rotarod (*N*), pole (*O*), beam (*P*), and locomotor activity test (*Q*) at 8 wk posttransplantation. Significant differences from WT mice at ^#^*P* < 0.05, ^##^*P* < 0.01, and ^###^*P* < 0.001 and from PD mice (PBS injection [sham-operated]) at *P < 0.05 and **P < 0.01, *n* = 6 (WT), 7 (PD and PBS), or 8 (PD and VM-Ast), one-way ANOVA, followed by Bonferroni post hoc analysis. (Scale bars: 50 μm in *B*, *D*, and *I* and 500 μm in *K*.)

Based on the in vitro data, our final mission was to test if cultured VM astrocytes could be utilized to treat α-synucleinopathy in vivo. Transplantation of cultured astrocytes is suggested as a useful therapeutic modality to treat neurodegenerative disorders (reviewed in ref. 74). Thus, PD model mice were generated by bilaterally injecting α-syn PFF + adeno-associated virus (AAV)-expressing α-syn into the SN (28). One month later, cultured VM astrocytes were transplanted into the right side of the SNs of the PD mice and α-synucleiopathy and DA neuron degeneration were compared with the contralateral (left) SNs of the same animals that underwent sham operation (phosphate-buffered saline [PBS]-injected) 2 mo after transplantation (3 mo after α-syn injection) (Fig. 5*A*). As previously described (28), there is substantial immunoreactivity for pS129-α-syn in the SNs of mice injected with the combined α-syn PFF+ α-syn-AAV2, which is indicative of pathologic α-syn aggregation (Fig. 5*D*). Along with an efficient engraftment of astrocytes (Fig. 5 *B* and *C*), pS129-α-syn immunoreactivity was significantly reduced in the right SNs of α-syn-PD mice grafted with cultured VM astrocytes (pS129-α-syn mean immunofluorescent intensity [MFI]: 22.3 vs. 17.1; percent pS129-α-syn+ of TH+ cells: 6.94 ± 1.25% vs. 4.05 ± 1.00% in the right vs. left SNs; Fig. 5 *D*–*F*). Compared with sham-operated SNs (left), TH+ DA neurons in the transplanted SN (right) looked much heathier, with larger cell bodies and longer neurite outgrowth (Fig. 5 *D*, enlarged images of boxed areas, Fig. 5 *G* and *H*). Furthermore, unbiased stereological counting showed more TH+ DA neurons in the right SNs grafted with astrocytes (4,266 ± 128 cells) compared to the sham-operated left SNs (3,045 ± 189 cells) (Fig. 5 *I* and *J*). Accordingly, nigrostriatal dopaminergic innervation was also significantly protected by astrocyte transplantation (TH+ fiber intensity estimated using ImageJ: 0.81 in the right vs. 0.56 in the left striatum, *P* < 0.01 on paired *t* test; Fig. 5 *K* and *L*). These findings collectively indicate that transplantation of cultured VM astrocytes prevents α-syn pathology and α-syn–mediated DA neuron degeneration.

Finally, to assess the effects of astrocyte transplantation on PD-associated behaviors, VM astrocytes (or PBS in the sham-operated control) were grafted bilaterally into the SNs of α-syn-PD model mice (Fig. 5*M*). Behavioral deficits manifested in the α-syn–injected mice 3 mo after α-syn injection. Compared to sham-operated α-syn PD mice, the behavioral deficits, as assessed by rotarod and pole tests, were significantly alleviated in mice transplanted with VM astrocytes (Fig. 5 *N* and *O*). In addition, impaired motor coordination (beam test) and hypo-locomotor activity (open field test) in the α-syn-PD mice showed a trend toward improvement with astrocyte transplantation (Fig. 5 *P* and *Q*).

**Fig. 6.**
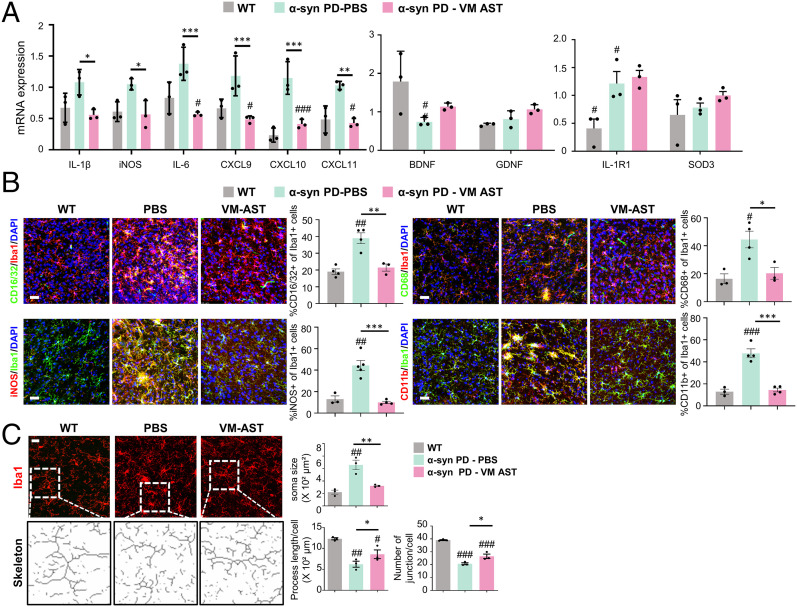
Improvement of host brain environment by grafting cultured VM astrocytes. Astrocytes cultured from the mouse VM were transplanted into the SN of α-syn-PD mice. Two months posttransplantation, local inflammation in the grafted SNs (host brain regions neighboring the grafts) was assessed compared with sham-operated (PBS-injected) SNs of α-syn-PD mice and WT mouse SNs. (*A*) qPCR data for proinflammatory, antiinflammatory, and neurotrophic cytokines. (*B*) Immunohistochemical analyses of microglia (Iba1+) immunoreactive for proinflammatory/cytotoxic factors (CD16/32, CD68, iNOS, and CD11b). Immunoreactive cells along the graft–host interfaces were counted in six cryosectioned slices from three animals in each group. Data are expressed as percentages of the immunoreactive cells of Iba1^+^ microglial populations. (*C*) Morphometric analysis of microglia (Iba1+). Soma size, process length, and number of junctions were assessed using LAS (soma size) and Analyze Skeleton plugin of ImageJ software (process) in a total 92 to 185 Iba1+ cells, randomly selected from three mouse SNs of each group. Significant differences from WT mice at ^#^*P* < 0.05, ^##^*P* < 0.01, and ^###^*P* < 0.001 and from PD mice (PBS injection [sham-operated]) at *P < 0.05, **P < 0.01, and ***P < 0.001. One-way ANOVA, followed by Bonferroni post hoc analysis. (Scale bar: 50 μm.)

The in vitro findings in *SI Appendix*, Fig. S6 *A* and *B* suggested that astrocyte action modulates microglial inflammation. Consistently, our previous study showed that transplantation of cultured astrocytes corrects local proinflammatory environments of the host brain (75). To further confirm astrocyte effects in an α-syn-PD context, the following qPCR and immunohistochemical experiments were designed. Messenger RNA expression of proinflammatory phenotype genes (IL-1β, iNOS, IL-6, CXCL9, CXCL10, and CXCL11) was greatly enhanced in the SNs after α-syn treatment (Fig. 6*A*). Proinflammatory gene expression was significantly down-regulated in α-syn–treated SNs (graft–host interfaces) by cultured VM-astrocyte grafting (Fig. 6*A*). In contrast, the expression of antiinflammatory phenotype markers (IL-1RN), neurotrophic factors (BDNF and GDNF), and antioxidant proteins (SOD3) was up-regulated in the transplanted SN (Fig. 6*A*). Furthermore, immunohistochemical analysis showed that the percentage of microglia expressing the proinflammatory factors CD16/32, CD68, iNOS, and CD11b in SNs treated with α-syn was 1.8- to 4.5-fold greater than in untreated wild-type (WT) SNs, but transplantation of cultured VM astrocytes greatly reduced this to WT levels (Fig. 6*B*). The astrocyte transplantation effect was further confirmed by the morphometric analysis demonstrating that inflated (hypertrophic) microglia, a characteristic of inflammatory reactive phenotype, in the sham-operated α-syn-PD mouse SNs, were restored to their homeostatic ramified morphology in the transplanted SN (Fig. 6*C*). These findings collectively suggest that the proinflammatory toxic environment and microglia phenotype were corrected by the astrocyte transplantation. Based on the α-synucleinopathy and DA neurodegeneration promoted in inflammatory environments (reviewed in ref. 46), the observed amelioration of α-synucleinopathy and DA neuron degeneration in the grafted SN is at least in part rescued by transplanted astrocyte-mediated correction of the local brain environment and microglial phenotype.

### Cografting VM Astrocytes Prevents Host-to-Graft α-Syn Propagation.

**Fig. 7.**
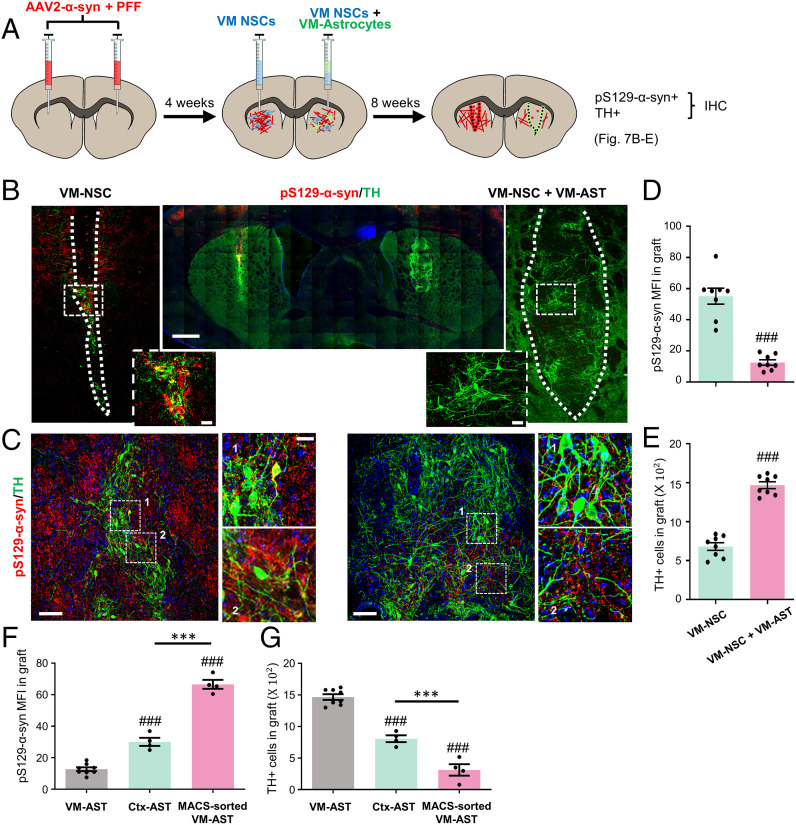
Host-to-graft transmission of α-syn pathology prevented by cografted astrocytes. (*A*) Schematic summary of the experimental procedure. α-synucleinopathic striatal environments were built by bilateral injection of the α-syn AAV2+PFF into the rat striatum. One month later, the dopamine neurogenic NSCs were transplanted into the left side of the α-synucleinopathic striatum, while the mixture of the NSCs with VM astrocytes, Ctx astrocytes, or ACSA-2-MACS–isolated astrocytes (2:1) was transplanted into the right striatum. (*B*–*E*) Effect of VM-astrocyte cografting on host-to-graft α-syn transmission and DA neuron engraftment. Representative images for pS129-αsyn+/TH+ grafts in the left and right striatum from two different rats (*B* and *C*). The boxed areas were enlarged in the neighboring images. pS129-αsyn expression in graft, estimated by MFI using LAS image analysis (Leica) (*D*). TH+ cells in graft (*E*). Significant differences from the left side of the striatum of identical animals at ^###^*P* < 0.001, *n* = 8 rats, paired *t* test. (Scale bars: 500 μm in *B*, 100 μm in *C*.) (*F* and *G*) Comparison of cografting effects of astrocytes from different origins. Host-to-graft α-syn propagation and DA neuron engraftment were assessed by the intensity of pS129-α-syn immunoreactivity (*F*) and the number of TH+ cells (*G*) in the grafts. Significant differences from the VM astrocyte cografted at ^###^*P* < 0.001 and between the groups indicated at ***P < 0.001, *n* = 8 (VM-Ast), 4 (Ctx-Ast), and 4 (MACS-sorted Ast); one-way ANOVA.

Given clinical experiences of fetal midbrain transplantation in PD, stem cell–based therapy has long been suggested as a potential disease-modifying therapy. In our previous study (75), we showed that cografting of cultured astrocytes enhanced NSC-based cell therapeutic outcomes with robust DA neuron engraftment and behavioral recovery in a PD animal model. This implies that cotransplantation of cultured astrocytes could be an ideal strategy for successful PD cell therapy. However, based on potential transmission of host α-syn to graft in the diseased brain of PD patients (19, 20), the prevention of host-to-graft α-syn propagation remains to be addressed. In vitro and in vivo observations described above indicate this α-synucleinopathy issue in the stem cell–based strategy could be solved using therapeutic functions of VM astrocytes. To test it, an α-syn pathologic environment was built in the striatum, the target site in PD cell therapy, by bilateral intrastriatal injection of combined α-syn PFF+ α-syn-AAV2 (Fig. 7*A*). Three months after the combined α-syn injection, α-synucleinopathy was severely disseminated over the striatal area with abundant Lewy body and neurite structures. NSCs with an efficient DA neurogenic capacity were prepared from the VMs of rat embryos (at E12). The VM-NSCs were transplanted into the left side of the α-synucleinopathic striatum, while the right striatum was grafted with a mixture of the VM-NSCs and VM astrocytes (2:1 in cell numbers). Under this harsh α-synucleinopathic condition, a few TH+ DA neurons were developed and detected in the left striatum grafted with the VM-NSCs alone at 2 mo posttransplantation (3 mo after α-syn injection) (Fig. 7 *B*–*D*). In addition, severe neuronal degeneration of TH+ DA neuronal cells with fragmented neurites was found (Fig. 7*C*). pS129-α-syn+ Lewy bodies and neurites were also abundantly detected in the left grafts but were greatly reduced in the right grafts generated by cotransplantation. In quantification, pS129-α-syn MFI was 55.1 in the left versus 12.5 in the right grafts (Fig. 7 *B*–*D*). Furthermore, consistent with the previous study (75), TH+ DA neurons in the right grafts (1,468 ± 44 cells) generated by cografting with VM astrocytes were much more abundant than in the left grafts (680 ± 49 cells) and exhibited healthier neuronal shapes with less-severe neurite degeneration (Fig. 7 *B*, *C*, and *E*).

Consistent with the in vitro findings above, cografting Ctx astrocytes resulted in decreased prevention of host-to-graft α-syn dissemination and DA neurodegeneration compared with that of VM astrocytes (Fig. 7 *F* and *G* and *SI Appendix*, Fig. S10 *A*–*C*). We further tested the cografting effect of astrocytes directly isolated from the adult mouse VM using magnetic-activated cell sorting (MACS) against the astrocyte-specific marker GLAST (ACSA-2). Cotransplantation of the MACS-sorted adult astrocytes did not improve a-syn pathology (pS129-α-syn+ bodies/neurites) or rather decreased DA neuron engraftment (TH+ DA neurons) in the grafts (*SI Appendix*, Fig. S10 *D*–*F*). These data were consistent with previous studies claiming that cultured astrocytes are immature, and cultured immature astrocytes, but not their counterparts in the adult brain, have therapeutic effects on transplantation (reviewed in ref. 74). However, we cannot exclude that the lack of therapeutic effects may be due to cellular stress accumulated in donor cells during cell dissociation and MACS procedures.

Collectively, these findings strongly suggest that the therapeutic functions of cultured VM astrocytes could be utilized both to treat endogenous α-synucleinopathy in the midbrain SN of PD and to prevent host-to-graft transmission of toxic α-syn in a stem cell–based cell therapeutic approach.

## Discussion

No therapeutic tool is currently available to address α-synucleinopathy. Based on our idea to develop therapeutic methods utilizing homeostatic brain functions, we scrutinized astrocytic functions to regulate a series of α-syn proteostasis processes in this study. We showed that astrocytes, especially those cultured from the VM, exert multifaceted therapeutic actions to prevent or alleviate α-syn pathology. In summary, pathologic α-syn aggregate formation was reduced by astrocytic paracrine action 1) to inhibit intraneuronal α-syn aggregation and 2) to regulate extracellular α-syn aggregation/disassembly. In addition, monomeric and aggregate forms of α-syn proteins were eliminated by 1) cell-autonomous astrocytic function to phagocytose and clear extracellular α-syn and 2) paracrine astrocytic action to stimulate neuronal autophagic clearance of intracellular α-syn.

Astrocytes in the brain exert physiologic roles to protect neurons from toxic extra- and intracellular environments in multiple ways, such as scavenging neuronal ROS (76), releasing neurotrophic factors and antiinflammatory cytokines (77), and transferring healthy mitochondria from astrocytes to damaged neuronal cells (78, 79). Intraneuronal oxidative/mitochondrial stress and extracellular brain inflammation were consistently corrected by molecules secreted from cultured astrocytes in this study. Thus, considering that misfolded α-syn aggregation and transmission are facilitated in stressful environments (40–42), the observed antisynucleinopathic functions of cultured astrocytes are likely mediated at least in part by their neurotrophic/antiinflammatory activities.

The naïve neurotrophic/antiinflammatory properties of astro cytes could explain why astrocytes derived from VM had a greater capacity to correct pathologic α-syn accumulation than those from the cortical brain region. Like neurons, astrocytes also have regional identities, and VM astrocytes are specified with the midbrain-specific factor expression (75). Among those, Nurr1 and Foxa2 have been shown to have the potential for glial antiinflammatory and neurotrophic functions (75, 80, 81). VM astrocytes expressing these midbrain-specific factors (*SI Appendix*, Fig. S1*C*) exert greater antiinflammatory/neurotrophic functions than cortical astrocytes (75). Thus, considering the positive link between neurotrophic/antiinflammatory and antisynucleinopathic capacities of astrocytes, the expression of midbrain-specific factors likely contributes to the superiority of VM astrocytes in the capacity to correct α-synuclein pathology.

In addition to the effect of correcting pathologic aggregated forms of α-syn, this study also identified astrocytic action to reduce total α-syn protein levels, partially by facilitating the neuronal autophagy process in a paracrine manner. The intracellular autophagy process is largely regulated by environmental factors (53). Considering that an astrocyte is the major cell type to establish brain environments surrounding neurons, the astrocytic roles that regulate neuronal autophagy were expected (reviewed in ref. 82), but they represent a nascent line of investigation. One potential paracrine molecule would be interferon-β, acting as a pro- or antiinflammatory cytokine that is highly expressed and secreted from cultured astrocytes, especially those cultured from VM (75). It has been reported that interferon-β promotes neuronal autophagy and α-syn clearance (53). Molecules and mechanisms underlying the paracrine effects of astrocytes remain to be further defined.

Another method for the astrocyte-mediated α-syn clearance we identified is the cell autonomous role of astrocytes to efficiently phagocytose extracellular α-syn aggregates and degrade them. This is in clear contrast to the way of treating exogenous α-syn fibrils by neuronal cells, in which misfolded α-syn aggregate formation grows over time after uptake of exogenous α-syn fibrils (*SI Appendix*, Fig. S9). The current hypothesis of Lewy body formation/propagation proposes that α-syn fibrils seed recruitment of endogenous soluble α-syn proteins and their conversion into insoluble pathological species(31, 59), indicating that endogenous α-syn expression is required for pathologic α-syn propagation. In contrast to neurons, endogenous α-syn expression is not significantly detected in astrocytes (11). In addition, it has been reported that autophagy in astrocytes is robustly activated upon metabolic stress, while it is less pronounced in neuronal cells (83), indicating the stronger capacity of astrocytes to clear toxic cellular debris and protein aggregates. The differences in proteotoxin clearance capacity as well as endogenous α-syn expression are suggested to contribute to the different modes of treating exogenous α-syn fibrils in astrocytes and neurons. Consistent with our observation, efficient astrocyte activity to phagocytose and degrade α-syn toxic species has also been demonstrated in recent literature (9–11). However, along with the detection of α-syn inclusions (glial cytoplasmic inclusions) in astrocytes of advanced PD and dementia with Lewy bodies brains (13–17), there is a contrasting view of the astrocyte roles in α-synucleinopathy, which proposes that astrocytes contribute to increasing neuronal α-synucleinopathy via uptake and subsequent prion-like propagation of the phagocytosed pathologic material (84). Considering that the presence of α-syn inclusion in astrocytes is usually detected in the advanced stage of the diseases when homeostatic astrocytes have been reactivated into the neurotoxic type of astrocytes, it is quite possible that the contrasting roles of astrocytes in neuronal α-synucleinopathy are determined by astrocyte reactivation status, such as beneficial effects by homeostatic/neurotrophic astrocytes (referred as to the A2 phenotype) in healthy brains or at early disease stages versus detrimental effects by neurotoxic astrocytes (A1 phenotype) at late and advanced disease stages.

The neurotoxic/detrimental polarizing potential of astrocytes raises concerns in therapeutic development in terms of directly transplanting astrocytes because grafted astroglia may possibly convert into the harmful phenotype after transplantation to a hostile diseased brain. However, cumulative studies have demonstrated that cultured astrocytes (as compared to the counterparts in an adult brain) exhibit immature properties (75, 85, 86). The transplanted immature astrocytes do not undergo harmful reactivation after a CNS injury but rather support neurite outgrowth and reduce glial scar formation in the injured CNS (reviewed in ref. 74). We have further shown that the toxic inflammatory brain environment is, rather, corrected by transplanting cultured astrocytes (ref. 75 and Fig. 6). Based on these findings, we attempted grafting with cultured astrocytes to treat α-synucleinopathy and showed the therapeutic effects in an α-synucleinopathic PD animal model.

In summary, we identified naïve astrocytic functions to correct α-syn pathology in this study and proposed transplantation of cultured VM astrocytes as a potential therapeutic option to treat α-synucleinopathy in PD. Further studies using clinically applicable cells like astrocytes of human origin such as those derived from human pluripotent stem cells (and with human mDA progenitor cells) and for a longer time course are needed. Aside from astrocyte transplantation, the observed paracrine effects of cultured astrocytes to control toxic α-syn aggregation, disassembly, transmission, and neuronal autophagic clearance in vitro strongly suggest a potential therapeutic development to treat α-synucleinopathy using soluble factors and exosomes secreted from cultured astrocytes. Our study has significant implications for astrocyte-based therapeutic development for α-synucleinopathic disorders.

## Materials and Methods

Astrocytes, microglia, NSCs, and mDA neurons were primarily cultured from mouse (ICR) and rat (Sprague-Dawley). An in vitro a-syn-PD model has been established in mDA neuron-enriched cultures with the combined human α-syn-lentivirus and PFF treatment. Alpha-syn-induced PD mice and rats were generated by the combined treatment of α-syn-AAV2 and PFF. Animals were housed and cared in accordance with NIH guidelines. All procedures for animal experiments were approved by the Institutional Animal Care and Use Committee at Hanyang College of Medicine under approval numbers 2019-0181A and 2019-0014A. The detailed methods for the cell cultures, α-syn PFF and virus preparations, α-syn proteostasis assays, PDI activity assay, WB, immunostaining, PCR, animal care, α-syn-PD-mouse modeling, astrocyte transplantation, histological analysis, behavioral assays, statistical analysis can be found in *SI Appendix*, *Supplementary Materials and Methods*.

## Supplementary Material

Supplementary File

## Data Availability

RNA-seq data from this study are available in the Gene Expression Omnibus (https://www.ncbi.nlm.nih.gov/geo/query/acc.cgi?acc=GSE106216) (87). All other study data are included in the article and/or *SI Appendix*.
