# Supplementary material for: Therapeutic functions of astrocytes to treat α-synuclein pathology in Parkinson’s disease
Source: Proc Natl Acad Sci U S A. 2022 Jul 15;119(29):e2110746119. doi: 10.1073/pnas.2110746119 (PMC9304026; doi:10.1073/pnas.2110746119)
Supplement: Supplementary File [file pnas.2110746119.sapp.pdf]

PNAS
www.pnas.org

Supplementary Information for
Therapeutic functions of astrocytes to treat α-synuclein pathology in Parkinson's disease

Yunseon Yang[1-3,*], Jae-Jin Song[1-3,*], Yu Ree Choi[4,5], Seong-hoon Kim[1-3], Min-Jong Seok[1-3], Noviana Wulansari[1-3], Wahyu Handoko Wibowo Darsono[1-3], Oh-Chan Kwon[1-3], Mi-Yoon Chang[1,2], Sang Myun Park[4,5 §], Sang-Hun Lee[1-3,§]

[§]Correspondence:
Sang-Hun Lee, M.D., Ph.D., Department of Biochemistry and Molecular Biology, College of Medicine, Hanyang University, 17 Haengdang-dong, Sungdong-gu, Seoul 133-791, Korea. Telephone: +82-2-2220-0625; Fax: +82-2-2220-2422;
E-mail: leesh@hanyang.ac.kr

Sang Myun Park, M.D., Ph.D., Department of Pharmacology, Ajou University School of Medicine, Suwon, Korea. Tel: +82-31-219-5063; Fax: +82-31-219-5069;
E-mail: sangmyun@ajou.ac.kr

This PDF file includes:

Supplementary text
Figures S1 to S10
SI References

Supplementary Materials and Methods

Cell culture

Primary astrocyte and microglia cultures

Astrocytes were cultured from the VMs and cortices of rat (Sprague-Dawley, SD) or mouse (ICR) at postnatal day 5 (P5) as previously described (1). Briefly, the brain tissues were dissected, triturated in complete medium (DMEM-F12 (Thermo Scientific, MA, USA) with 10% FBS, 10% horse serum (HyClone, GE Healthcare Life Sciences, PA, USA), 1 mM L-glutamine (Life Technologies, CA, USA), and 1% PenStrep (Thermo Scientific). The cell suspension was then seeded and cultured in a T75 flask (Thermo Scientific). At day 14, astrocytes attached to the culture surface were separated from floating microglia by shaking the flask on an orbital shaker at 0.4 x *g* for 12 hrs at 37°C. The astrocytes and microglia harvested were sub-cultured on a culture surface coated with poly-L-ornithine/fibronectin (Millipore Sigma, MO, USA) for 5-20 days.

NSC culture

NSCs with mDA neurogenic potential were cultured from VM of mouse (ICR) embryos at E10.5 or rat (Sprague-Dawley) embryos at E12 as previously described (2). The VM-NSCs were expanded in N2 medium supplemented with basic fibroblast growth factor (bFGF, 20 ng/ml; R&D Systems, MN, USA) and epidermal growth factor (EGF, 20 ng/ml; R&D Systems) for 3-6 days. For differentiation, mitogens (bFGF and EGF) were withdrawn in N2 medium. Glial populations were eliminated by adding Ara-C (3 μM; Sigma) during differentiation days 3–5. NSCs were also cultured from the cortices, a nondopaminergic brain region (mouse at E12.5 or rat at E14), and used as control cells for the cultured astrocytes.

MACS-based isolation of astrocytes from mouse VM

VMs, dissected from mice (ICR) at postnatal day 14, were incubated at 37°C in 0.05% Trypsin-EDTA+HBSS solution (1:9, v/v) for 30 minutes, and dissociated carefully. Astrocytes were isolated from the cell dissociates by magnetic activated cell sorting (MACS) using an Anti-ACSA-2 Microbead kit (Miltenyi Biotec, Cologne, Germany)

following the manufacturer's protocol. Astrocytes separated were used directly for co-transplantation experiment.

CM preparation and treatment

Fresh N2 medium or DMEM (phenol red free) was added to cultured Ctx-astrocytes, VM-astrocytes, and Ctx-NSCs (control), and the CM was collected every other day for 4–6 days. The CMs were adjusted to 0.1–0.15 mg of protein/ml, filtered at 0.45 μm, and stored at −80°C until use. The CMs were diluted with N2 medium (1:1, v/v) before being adding to the cells in culture.

Preparation of α-syn PFF

Recombinant α-syn (human) and α-syn PFF were prepared as described previously (3). Briefly, 5 mg/mL monomeric α-synuclein was incubated at 37°C with continuous agitation at 1,000 rpm for 7 days, sonicated on ice for 3–5 sec at 3 W using an ultrasonic processor VC 505 (Sonics & Materials, Inc., CT, USA) and stored at −80°C until use as α-syn PFF. The status of α-syn fibrils was determined using the thioflavin T binding assay and transmission electron microscope (TEM, Zeiss, Oberkochen, Germany) observations.

In vitro culture to assess astrocyte function in α-synucleinopathy

To induce α-syn pathology in the cultured mDA neurons, the VM-NSCs were transduced with human α-syn-expressing lentiviruses and differentiated for 7–10 days. The human α-syn-overexpressing mDA neurons were co-cultured with Ctx-, VM-astrocytes (or Ctx-NSCs) using a culture insert device (Thermo Scientific) or directly mixing the cells at a 2:1 ratio of α-syn-mDA neurons to astrocytes, or cultured in the presence or absence of the conditioned media prepared from astrocytes (ACM) or Ctx-NSCs (C-CM). The cultures were treated with α-syn PFF (final concentration: 1-2 μg/ml) for 14–20 days with gradual dilution of PFF by changing half of the medium every other day.

Thioflavin T assay

α-syn PFF or monomer was incubated with ACM (C-CM or PBS as control) at 37°C. In certain experiments, the PDI inhibitors Bacitracin (0.1-5uM, Sigma) and DTNB (0.1-5uM, Sigma) were added to the incubation mixtures. Samples were collected and loaded in a 96-well black plate containing a 2 μM ThT solution. After shaking for 5 seconds in the plate reader, fluorescence intensity was measured at 450 nm (excitation) and 482 nm (emission).

Assay for PDI activity

A PROTEOSTAT® PDI assay kit (ENZ-51024-KP050, Enzo Life Sciences, NY, USA) was used to estimate PDI (protein disulfide isomerase) enzymatic activity. Conditioned media of Ctx-NSCs, Ctx-Astrocytes and VM-Astrocytes were collected with phenol red (-) media and concentrated 10 times using Vivaspin^{R} 20 (Viva Products, MA, USA). Experiments were performed following the manufacturer's protocols.

BiFC assay

VM-NSCs were co-transduced with lentiviruses expressing Venus1-α-syn (V1S; N-terminal of α-syn), α-syn-Venus2 (SV2; C-terminal of α-syn) and differentiated to mDA neurons. BiFC-α-syn aggregates were visualized as a Venus positive signal.

Western Blot analysis

Proteins in cell lysates were extracted using a RIPA buffer containing a protease inhibitor (Roche, Mannheim, Germany) and phosphatase inhibitor cocktail (Sigma). To detect aggregate forms of α-syn, protein was extracted using a 1%Triton X-100/PBS buffer containing protease inhibitor (Roche) and phosphatase inhibitor cocktails (Sigma). After centrifugation (16,000g, 10 minutes), Triton X-100 supernatant was collected (Triton X-100 soluble protein). The pellet was dissolved in 1% SDS sample buffer and then briefly sonicated (Triton X-100 insoluble protein). Protein concentrations were measured using the Pierce BCA Protein Assay Kit (Thermo Scientific). To detect proteins secreted from cultured cells, proteins in the culture media were concentrated by the methanol /chloroform precipitation method (4). Samples (15–25 ug of protein) were electrophoresed on SDS-PAGE gel (12% or 4–16% for α-syn aggregate detection),

transferred to a nitrocellulose (or polyvinylidene fluoride) membrane, blocked, and then incubated with primary antibodies. Primary antibodies used were anti-mouse α-syn (610787, BD biosciences), pS129-α-syn (825701, Bio Legend), caspase-1 (p20) (AG-20B-0042-C100, AdipoGen Life Sciences, CA, USA), β-actin (MA1-140, Invitrogen), anti-rabbit LC3 (NB100-2220, Novus Biologicals, CO, USA), p-62 (PM045, MBL, MA, USA), and anti-goat IL-1β (AF-501-NA , R&D Systems), PDI (ab2792, Abcam, Cambridge, England). Signals were visualized with horseradish peroxidase–conjugated antibodies and captured with ChemiDoc (Bio-Rad, CA, USA). Densitometric quantification of the bands was performed using ImageJ (Image Processing and Analysis in Java, NIH).

Assays for mitochondrial and oxidative stress

Mitochondrial ROS was estimated using MitoSox (Thermo Scientific) based on the manufacturer's protocol. Intracellular ROS levels were determined by staining with the ROS indicator 5-(and-6)-chloromethyl-20,70-dichlorodihydrofluorescein diacetate [CM-H2DCF-DA, DCF) (Life Technologies, CA, USA)].

Immunostaining

Cultured cells or cryosectioned brain slices were fixed with 4% paraformaldehyde in PBS, blocked in 0.3–0.6% Triton X-100 with 1% bovine serum albumin for 40–60 minutes, and then incubated with primary antibodies overnight at 4°C. For α-syn and pS129- α-syn immunostaining, samples were fixed with 4% paraformaldehyde/4% sucrose/1% Triton X-100 in PBS, blocked in 0.1% Triton X-100 with 3% bovine serum albumin for 15 minutes. The primary antibodies used were anti-mouse α-syn (610787, BD biosciences), pS129-α-syn (825701, Bio Legend), Gfap (691102, MP Biomedicals, CA, USA), Glast (130-095-821, Miltenyi Biotec), Cd44 (NBP1-31488, Novus Biologicals), S100β (S-2532, Sigma), Sox2 (AB5603, EMD Millipore corp); anti-rabbit TH (P40101-150, Pel-Freeze), Tuj1 (802001, Bio Legend), Gfap (Z0334, Dako), and Glt-1 (ab106289, Abcam), Cd68 (MAB1435, Sigma-Aldrich), Cd16/32(553142, BD biosciences), Inos(610431, BD biosciences), Cd11b(CBL1512, Sigma-Aldrich), Iba1(019-19741, Wako). Secondary antibodies tagged with Cy3 (1:200, Jackson Immuno

Research Laboratories, PA, USA) or Alexa Fluor 488 (1:200, Life Technologies) were used for visualization. The stained cells (brain sections) were mounted using VECTASHIELD with a DAPI mounting solution (Vector Laboratories, CA, USA), and images were obtained with an epifluorescence microscope (Leica, Heidelberg, Germany) and confocal microscope (Leica PCS SP5). For stereological counting of TH+ DA neurons in the mouse SN, peroxidase (HRP)-based colorimetric staining was applied using Vectastain ABC and DAB kit (Vector Lab). Fiber lengths and soma sizes of TH+ mDA neurons and the immunointensity of pS129-α-syn in brain slices were measured using an image analysis system (Leica LAS).

Real-time PCR

Total RNA was prepared using the Trizol reagent (Invitrogen, CA, USA) through the RNA isolation protocol. cDNA synthesis was carried out using a Superscript kit (Invitrogen). Real-time PCR was performed on a CFX96TM Real-Time System using iQTM SYBR green supermix (Bio-Rad, CA, USA), and gene expression levels were determined relative to GAPDH levels.

Phagocytosis and α-syn clearance assays

Phagocytic activities to uptake latex beads (FluoSpheres Polystyrene Microspheres), α-syn PFFs labeled with pHrodo Red or Alexa 488 (all from Invitrogen) were estimated in cultured Ctx-Astrocytes, VM-Astrocytes, and Ctx-NSCs (control). α-syn-PFFs were labeled with pHrodo (P36600) by incubating in 0.1 M bicarbonate buffer (pH8.4) for 1 hr. The cells (4×10^4 cells/well) were treated with latex beads (4×10^6 beads/μl, 12hrs) and pHrodo-labeled α-syn-PFFs (1 μM, 24hrs) and washed. Then, intracellular beads and red fluorescent+ cells were counted. Labeling of α-syn-PFFs with Alexa 488 was carried out using the protein labeling kit (A30006, Invitrogen) following the manufacturer's protocols. To estimate the α-syn clearance activity in cultured astrocytes, cells were treated with Alexa Fluor 488-labeled α-syn PFFs (1 μM, 3 days) and washed. Remaining intracellular fluorescent puncta were counted at multiple time points for 3–7 days.

Animal housing

All procedures for animal experiments were approved by the Institutional Animal Care and Use Committee at Hanyang College of Medicine under approval number 2019-0181A, 2019-0014A. Mice were housed in a specific pathogen-free barrier facility with a 12-h light/dark cycle and maintained on standard chow (5053 PicoLabR Rodent Diet 20). Experiments were performed in accordance with NIH guidelines.

Astrocyte transplantation to α-syn-PD rodent model

α-syn-induced PD mice (ICR) were generated by combined treatment with AAV2 expressing human α-syn (α-syn-AAV2) and α-syn-PFFs (5). Briefly, female mice were anesthetized by Zoletil50 (0.1 mg/kg) mixed with Rompun (93.28–l g/kg). α-syn-PFF (2 μl, 5 μg/μl in PBS) and α-syn-AAV2 (2 μl, 2.1×10^{12} GC/ml) were injected bilaterally into the SN (3.3 mm posterior to bregma; ±1.2 mm lateral to midline; − 4.6 mm ventral to dura) of mice (10 weeks age). The infusion was performed at a rate of 0.25 μl per min. The needle (26 gauge) was left in the injection site for 25–30 min after completion of each injection and then removed slowly. One month later, astrocytes cultured from mouse VM (3 μl, 1.5×10^5 cells/μl) were injected over a 10-minute period into the SN of the PD model mice (3 μl of PBS was injected for the sham control). To evaluate the effect of astrocyte co-grafting on host-to-graft α-syn transmission, the α-syn-AAV2+PFF was injected to the striatum (0.0, +/-0.3, -0.5; incisor bar set to 3.5 mm below zero) of rats (SD, 10 weeks). One month later, dopaminergic NSCs (derived from rat VM at E12) were mixed with VM-, Ctx- (derived from rats at P5) or MACS-sorted astrocytes (derived from mouse VM at P14) at a 2:1 ratio, and the mixed cells (3 μl, 1.5×10^5 cells/μl) were injected over a 10-minute period into the right side of the α-syn-injected striatum, while the left side of the striatum was grafted with the VM-NSCs alone (2 μl, 1.5×10^5 cells/μl + 1 μl, PBS). Animals received daily injections of cyclosporine A (10 mg/kg, i.p.) starting 1 day before the grafting and continuing for 3 weeks. They were maintained without the immunosuppressant for the rest of the post-transplantation period. Histological analyses were carried out 2 months post-transplantation.

Behavior test

All behavioral experiments measuring motor dysfunction were performed twice after 2 – 3 days of training.

Pole test

The animal was placed on top of a vertical wooden pole (50 cm in length, 1 cm in diameter) with the head toward the bottom. The time that the animal spent descending the length of the pole was measured. If the animal fell or slipped from the pole, it was given the highest value of the week.

Beam test

The motor coordination and balance of the animal were analyzed. Briefly, the animal was placed on a beam (square, 80 cm in length, 12 mm in diameter, 50 cm above the bottom) and the time required to cross the narrow beam was measured. If the animal fell from the beam, it was given the highest value of the week.

Rotarod test

To evaluate motor coordination, the animal was placed on a rotating rod with accelerated rotation (4–44 rpm speed, 300 seconds). The time that the animal stayed on the rotating rod was measured.

Locomotion test

The locomotor activities of the PD mice were monitored using a camera (HD C310, Logitech). Briefly, mice were placed in the center of a 40-cm square cage and allowed to freely explore the apparatus for 20 min while being tracked by a video-recording system. After each test, each mouse was returned to its home cage, and the open field was cleaned with 70% ethyl alcohol and permitted to dry. To assess the process of habituation to the novel arena, mice were then exposed to the apparatus for 20 min on two consecutive days for statistical analysis. Total distance and average speed were measured automatically by the software (Stoelting Co., IL, USA).

Histological analysis

For immunohistochemistry, animals were perfused intracardially with 4% paraformaldehyde in PBS. Brains were removed and immersed in 30% sucrose in PBS until they sank. SN and striatal regions were sliced into 30–40 µm thicknesses on a freezing microtome (CM 1850; Leica, Wetzlar, Germany) and subjected to immunofluorescence staining. The number, fiber length, and soma size, and pS129-α-syn immunoreactivities of TH+mDA neurons in the right sides of the SN (VM-astrocyte-grafted) and the striatum (VM-NSC+astrocyte-co-grafted) were compared with those of their left side (PBS-injected SN or VM-NSC-grafted striatum). The number of immunoreactive cells was counted via unbiased stereological counting (for TH+ DA neurons in the mouse SN) using a computer-assisted image analysis system consisting of an Axiophot photomicroscope (Carl Zeiss Vision) equipped with a computer-controlled motorized stage (Ludl Electronics), a Hitachi HV C20 camera, and Stereo Investigator software (MicroBright-Field) or manual counting with Abercrombie correction to compensate for double counting in adjacent sections (Abercrombie correction factor ($N = n \times T/(T+D)$), where N is actual number of cells, n is the number of nuclear profiles, T is the section thickness, and D is the average diameter of nuclei). The histological analyses were made every 8 sections throughout the midbrain and striatal grafts.

Statistical analysis

All data are expressed as the mean ± SEM, and statistical tests are justified as appropriate. The normality of data distribution was confirmed using the Shapiro-Wilk test at $p>0.05$. Statistical comparisons were made using the paired t-test, Student's t-test (unpaired) or one-way analysis of variance (ANOVA) followed by Bonferroni post hoc analysis using SPSS (Statistics 21; IBM Inc., AR, USA). The n-, p-values, and statistical analysis methods are indicated in the figure legends.

Supplementary Figure and Figure legends

Suppl. Fig S1

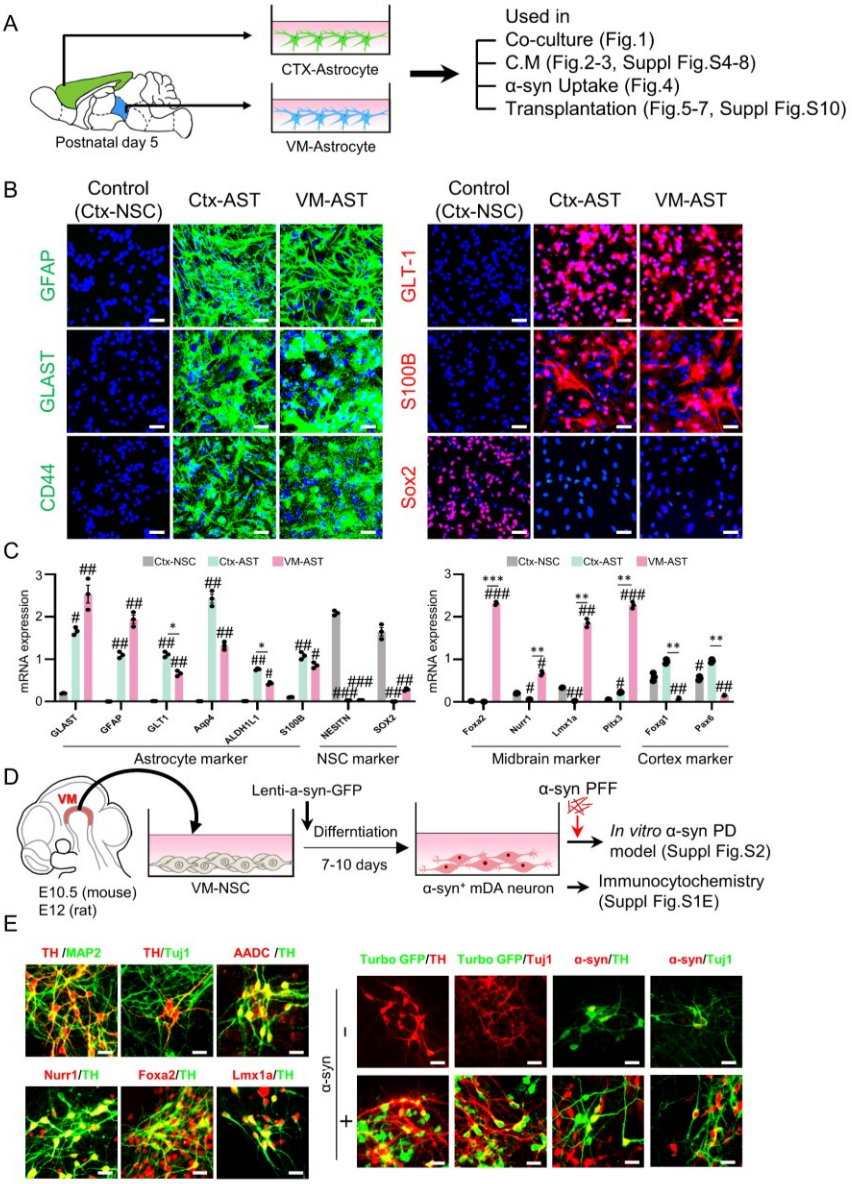

Suppl. Fig. S1. Preparation of astrocyte and α-syn-expressing midbrain dopamine (mDA) neuron-enriched cultures used in this study. A-C, Astrocytes cultured from mouse (rat) ventral midbrain (VM) and cortex (Ctx) at post-natal day 5. Expression of astrocytic markers and VM-, and Ctx-region-specific markers in the astrocyte cultures were estimated by immunocytochemical (**B**) and real-time qPCR (**C**) analyses. Undifferentiated neural stem/precursor cell (NSC) culture derived from mouse embryonic Ctx was used as the control. Data are presented as mean±SEM. Significant differences from the control Ctx-NSC cultures at $p<0.05^{\#}$, $p<0.01^{\#\#}$, $p<0.001^{\#\#\#}$, and from Ctx-

Astocytes at $p < 0.05$*, $p < 0.01$**, $p < 0.001$***, n=3, One-way ANOVA, followed by Bonferroni post hoc analysis. Scale bars are 20 μm. **D** and **E**, Preparation of α-syn-overexpressing mDA neuron culture. Dopaminergic NSCs derived from the rodent embryonic VM tissues at E10.5 (mouse) and E12 (rat) were in vitro expanded and transduced with lentiviruses expressing human α-syn labeled with GFP. The NSCs were differentiated to mDA neurons (**D**). Dopaminergic neuronal and midbrain-specific phenotypes and α-syn expression in the differentiated cultures were estimated using immunocytochemical analysis (**E**). Scale bars are 50 μm.

Suppl. Fig S2

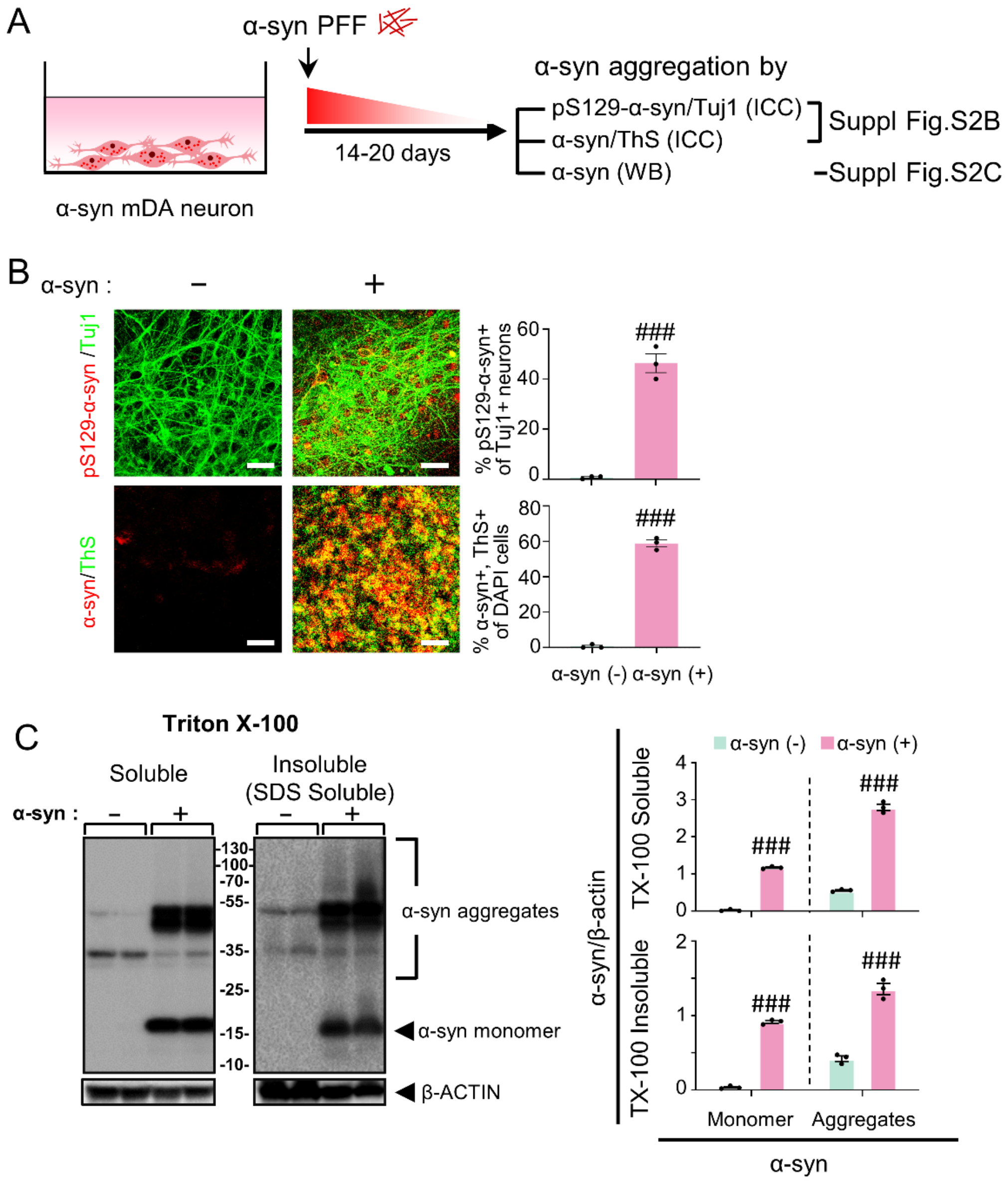

Suppl. Fig. S2. Establishment of in vitro cellular model for α-synucleinopathy in PD. Human α-syn-overexpressing mDA neuron cultures (prepared in Suppl. Fig. S1D-E) were treated with α-syn preformed fibril (α-syn-PFF) for 14-20 days with half of the α-syn PFF-containing N2 medium replaced with flesh N2 medium at a 2-days interval (**A**). α-syn aggregate formation was detected by % neuronal cells immunoreactive to pS129-αsyn, those stained with Thioflavin S (**B**), and WB analysis for α-syn (**C**). Significantly different from the α-syn-untreated control at $p<0.001^{\#\#\#}$, n=6 (**B** and **C**), Student's t-test, scale bar is 25 μm.

Suppl. Fig S3

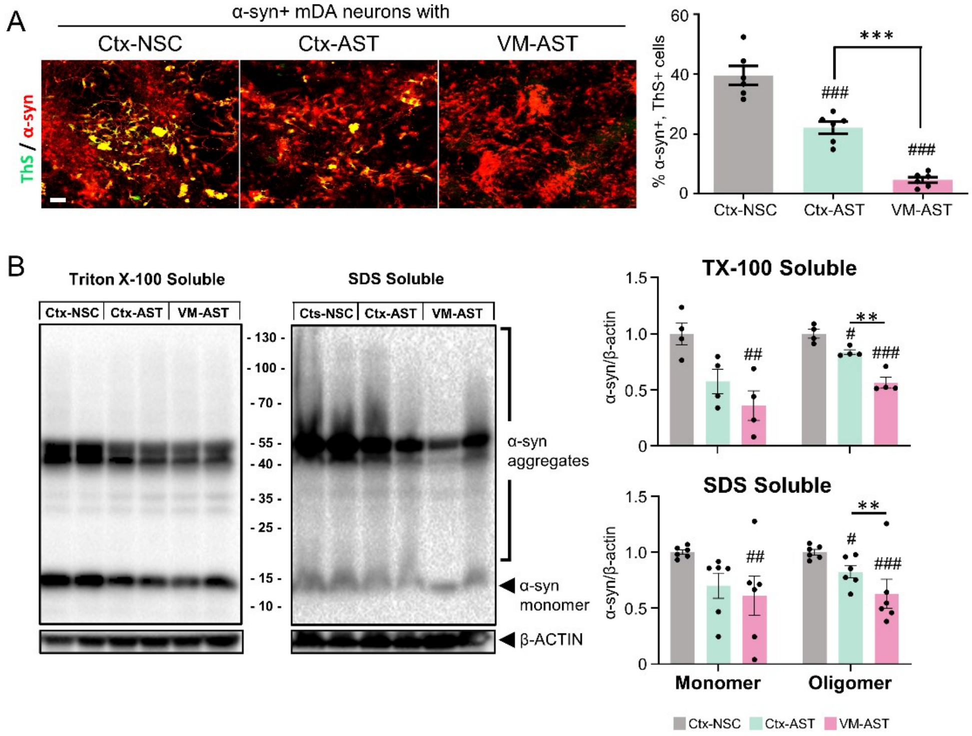

Suppl. Fig. S3. Astrocyte effects on neuronal α-synucleinopathy evaluated in the co-cultures where cultured neurons are directly mixed with cultured Ctx-Astrocytes, VM-Astrocytes or Ctx-NSCs (control). Neurons were mixed with the astrocytes (or Ctx-NSCs) at a 2:1 ratio. Neuronal α-syn aggregates were detected using % Thioflavin S+, α-syn+ cells (**A**) and α-syn western blot analysis (**B**). Significant differences from Ctx-NSC at $p<0.05^{\#}$, $0.01^{\#\#}$, $0.001^{\#\#\#}$ and between the groups indicated at $p<0.01$** and 0.001***, n=6 (A and B), One-way ANOVA, scale bars are 25 µm.

Suppl. Fig S4

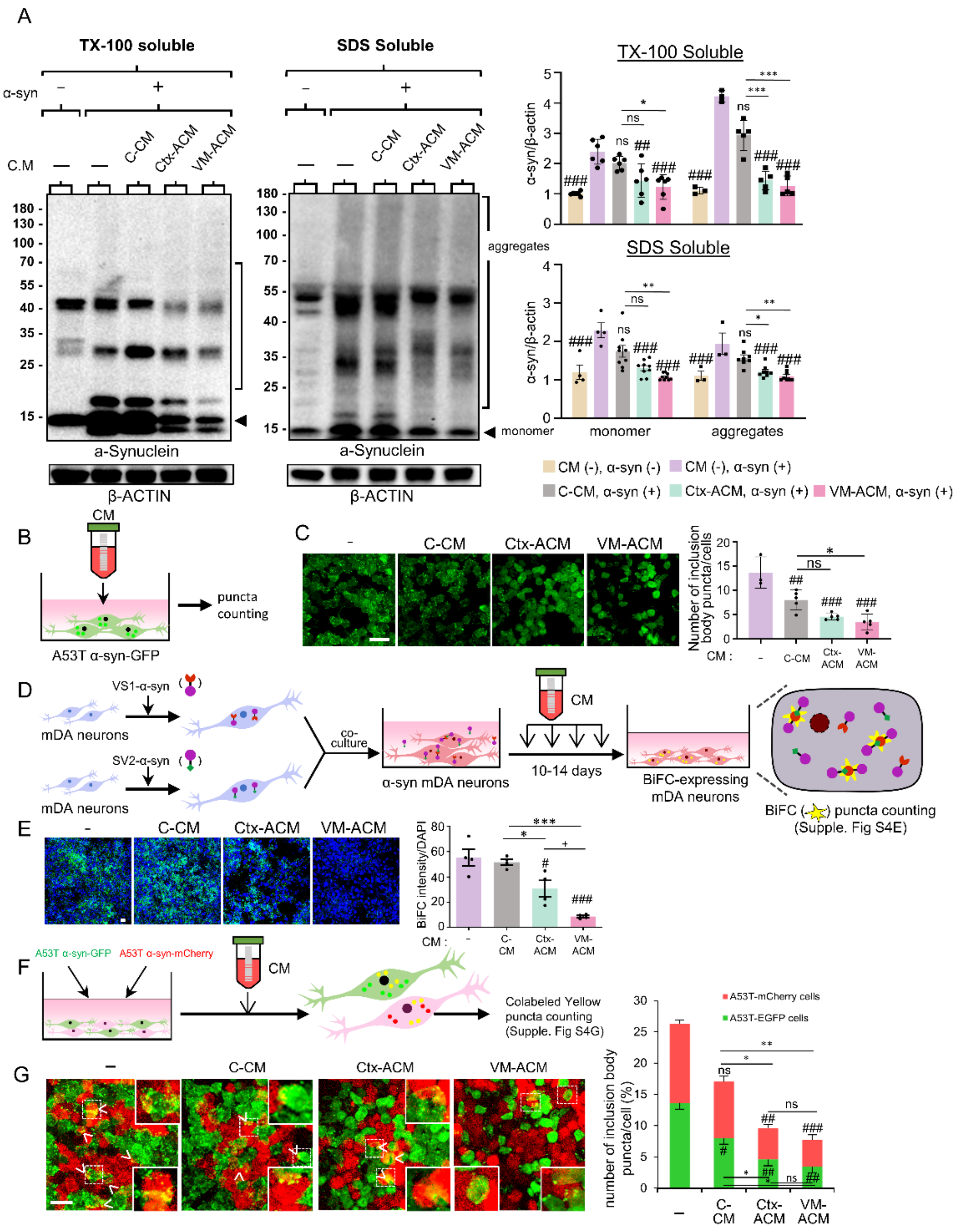

Suppl. Fig. S4. Astrocyte paracrine functions to alleviate α-syn aggregation and transmission. A, ACM effect on the levels of α-syn aggregates and monomers detected by WB analysis. **B-C**, Intra-neuronal α-syn oligomerization assessed by α-syn-GFP puncta counts in GFP-labeled A53T-α-syn-expressing SH-SY5Y neuronal cells. **D-G**, Combined α-syn transmission + oligomerization activity determined using the modified BiFC (**D-E**) and the co-cultures for GFP- and mCherry-labeled A53T-αsyn-expressing

SH-SY5Y cells (**F-G**). Significant differences from the α-syn-treated (+) and CM-untreated (-) control at $p<0.05^{\#}$, $0.01^{\#\#}$, $0.001^{\#\#\#}$ and between the groups indicated at p<0.05*, 0.01**, 0.001***, n=3-9, One-way ANOVA, scale bars are 25 μm.

Suppl. Fig S5

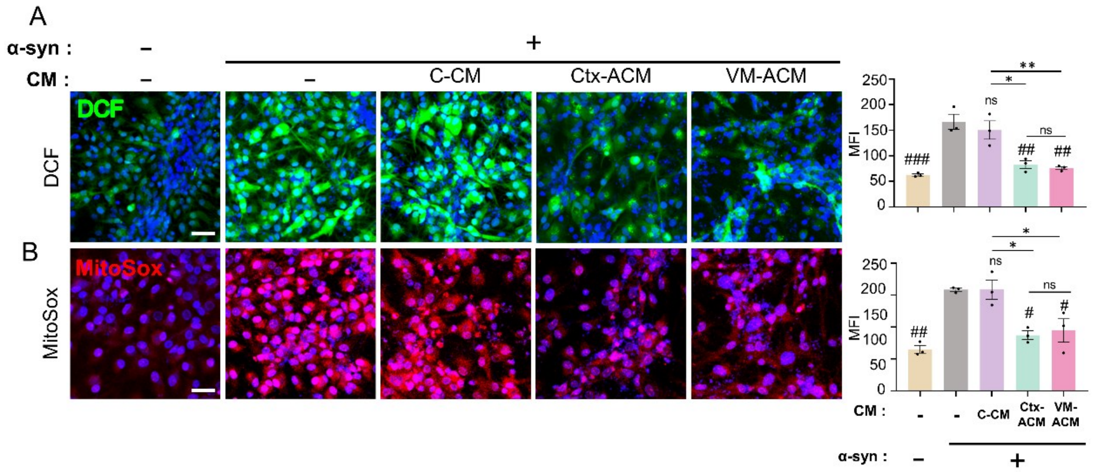

Suppl. Fig. S5. Factors secreted from astrocytes correct intra-neuronal oxidative and mitochondrial stresses. A, Intracellular ROS levels estimated by DCF-DA in mDA neuron-enriched cultures. **B**, Mitochondrial stress estimated by mitochondrial ROS levels using the MitoSox red probe. Fluorescence intensity was measured using ImageJ and presented as mean fluorescent intensity (MFI). Significant differences from the α-syn-treated (+) and CM-untreated (-) control at $p<0.05^{\#}$, $0.01^{\#\#}$, and $0.001^{\#\#\#}$ and between the groups indicated at p<0.05* and 0.01**, n=3 independent cultures, One-way ANOVA, scale bars are 25 μm.

Suppl. Fig. S6

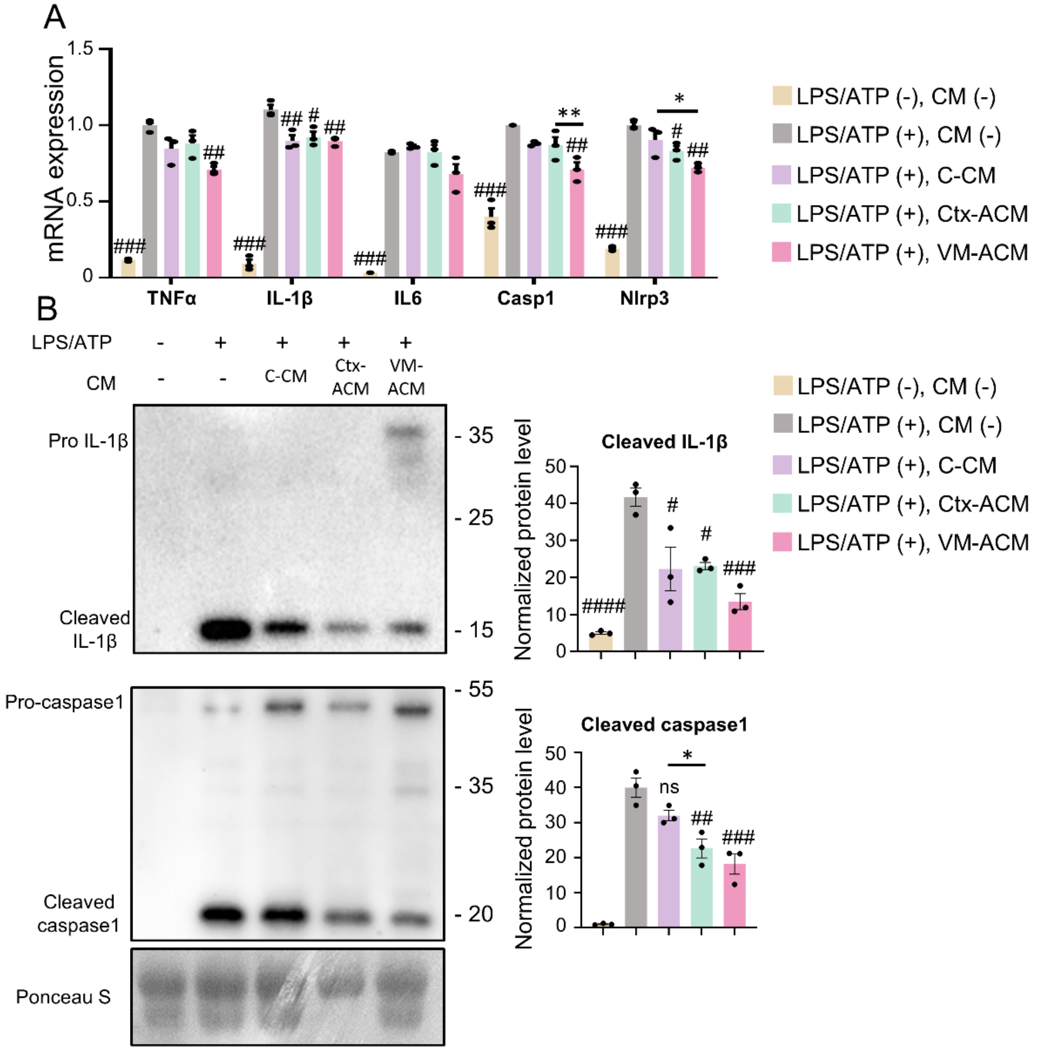

Suppl. Fig. S6. Astrocytes inhibit pro-inflammatory cytokine expressions and NLRP3-inflammasome activation in a paracrine manner. Primary microglia were treated with LPS (1 µg/ml, 4 hrs), followed by ATP treatment (2 mM, 30 min) in the presence or absence of CM. **A**, Real time-PCR analysis for Pro-inflammatory cytokine expression in cultured microglia. **B**, NLRP3 inflammasome activation assessed by WB-based measurement of the secreted protein levels of cleaved/activated forms of caspase-1 and IL-1β. The secreted protein levels were measured in the media and normalized by Ponceau S-stained total protein levels. Significant differences from the LPS+ATP-treated(+)/CM-untreated(-) at $p<0.05^{\#}$, $0.01^{\#\#}$, and $0.001^{\#\#\#}$ and between the groups indicated at p<0.05* and 0.01**, n=3-7 independent cultures, One-way ANOVA.

Suppl. Fig S7

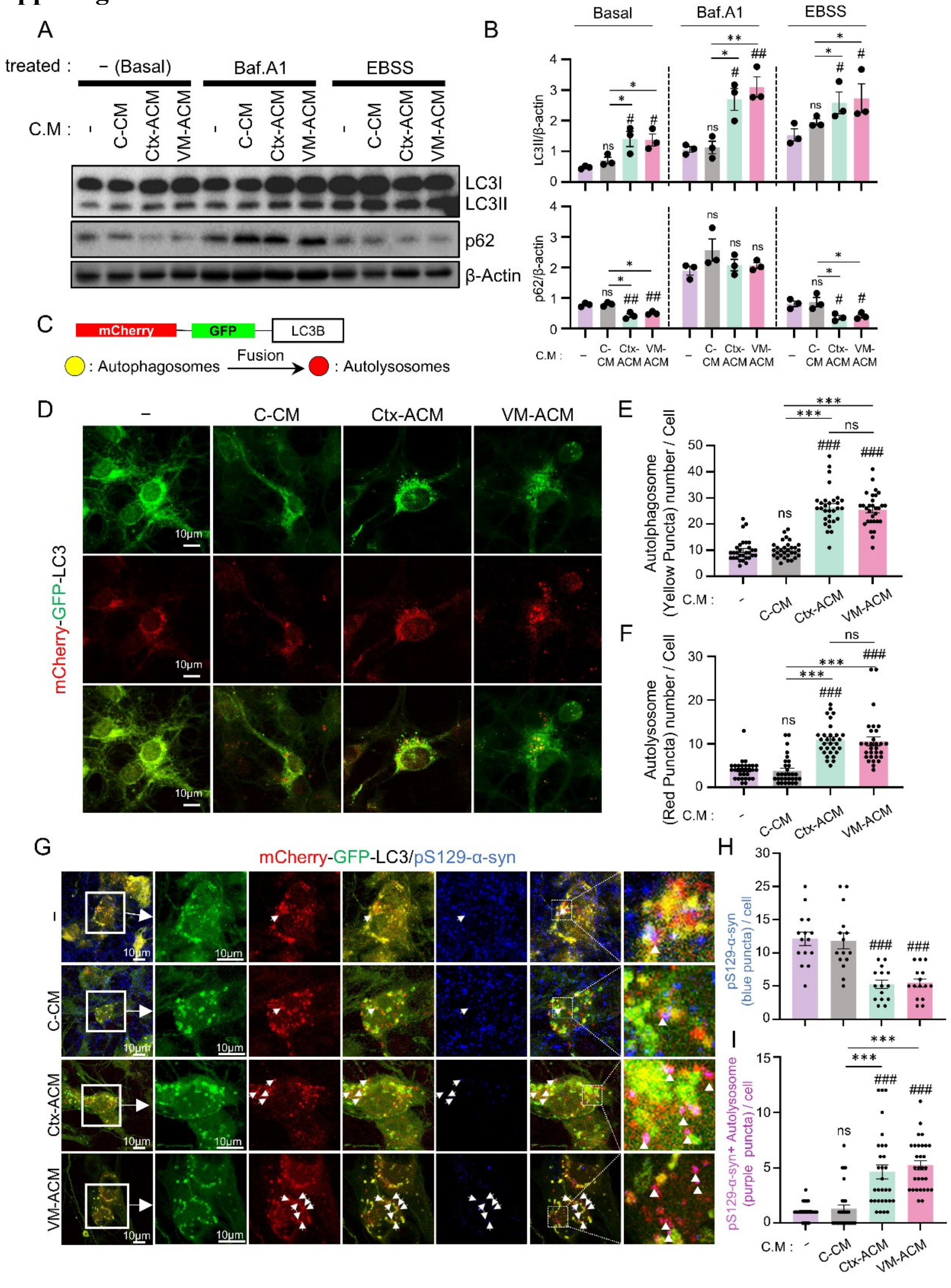

Suppl. Fig. S7. Astrocytes enhance neuronal autophagy in a paracrine manner. A-B, Western blot analysis of autophagosome component LC3(II) and p62 in the basal condition and in the presence of Bafilomycin A1 and/or EBSS treatment. Intensities of the bands in (**A**) were quantified using ImageJ software, and the values were normalized

to β-actin (**B**). n= 3 independent experiments. **C-D**, Representative image of differentiated neuron expressing mCherry-GFP-LC3B cultured in the presence or absence of conditioned medium. **C**, Schematic representation of the tandem LC3 reporter system. The processed LC3 reporter localizes to autophagosomes fluoresces both red and green. Upon fusion with the acidic lysosome, the low pH of the autolysosome quenches the GFP fluorescence, but the mCherry fluorescence remains stable. **E-F**, Quantification of autophagosomes (yellow puncta) (**E**) and autolysosomes (red puncta) (**F**) number per cell. n = 30 cells from each group. **G**, Representative images of pS129-αSyn staining in differentiated neuron expressing mCherry-GFP-LC3B, cultured in the presence or absence of conditioned medium. **H,** Quantification of pS129-αSyn+ puncta number per cell. n = 15 cells from each group. **I,** Quantification of pS129-αSyn+ puncta colocalized with autolysosome (red puncta) per cell. n = 15 cells from each group. Data are represented as means ± SEM. Significant differences from the CM-untreated (-) control at $p<0.05^{\#}$, $0.01^{\#\#}$, and $0.001^{\#\#\#}$ and between the groups indicated at p<0.05*, 0.01**, and 0.001***, two-way ANOVA followed by Bonferroni's post hoc test; ns, no significance.

Suppl. Fig S8

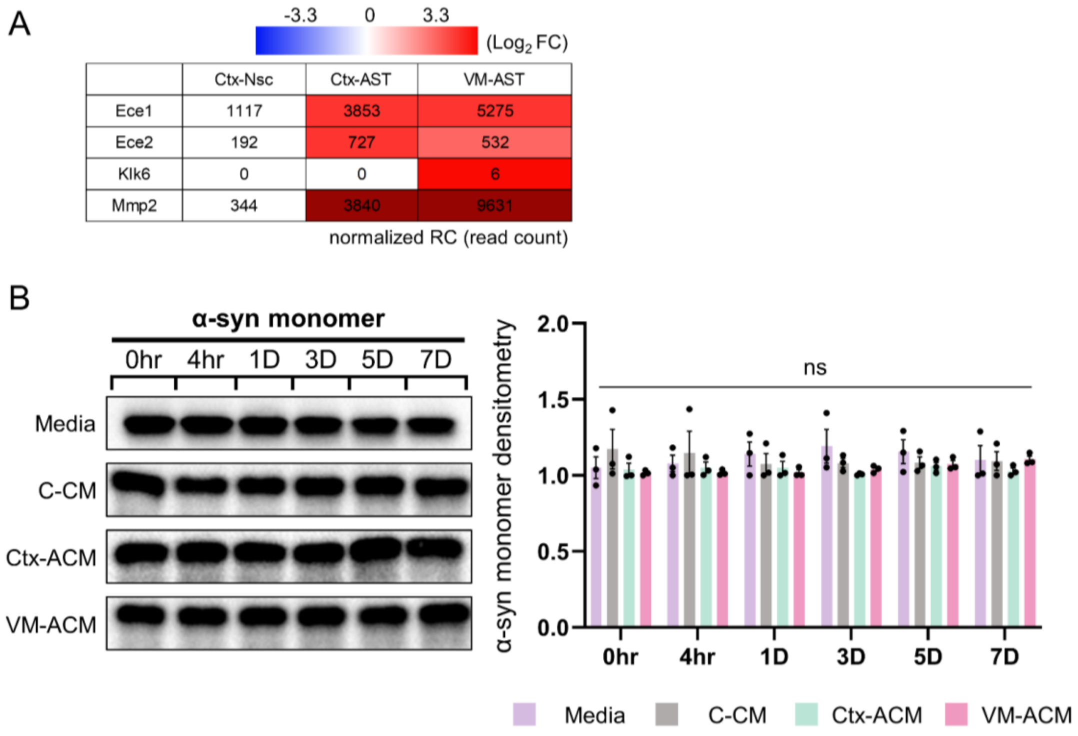

Suppl. Fig. S8. ACM activity for extracellular α-syn protein degradation in a cell-free condition. A, mRNA expressions of the secretory protein with the reported activity to degrade extracellular α-syn protein in the RNA-seq data (accession no. GSE106216). The RNA-seq data represent normalized read counts (RC, inside box) and Log2[Astrocyte/control Ctx-NSC](color intensities). **B**, WB data demonstrating time-course changes of α-syn protein levels in the presence of ACM. Monomer form of α-syn (500 μg) was incubated with the ACM or C-CM or culture medium (the controls) at 37°C for 7 days. Alpha-synuclein protein levels in aliquots of the reaction were monitored. ns, no significance, n=3.

Suppl. Fig S9

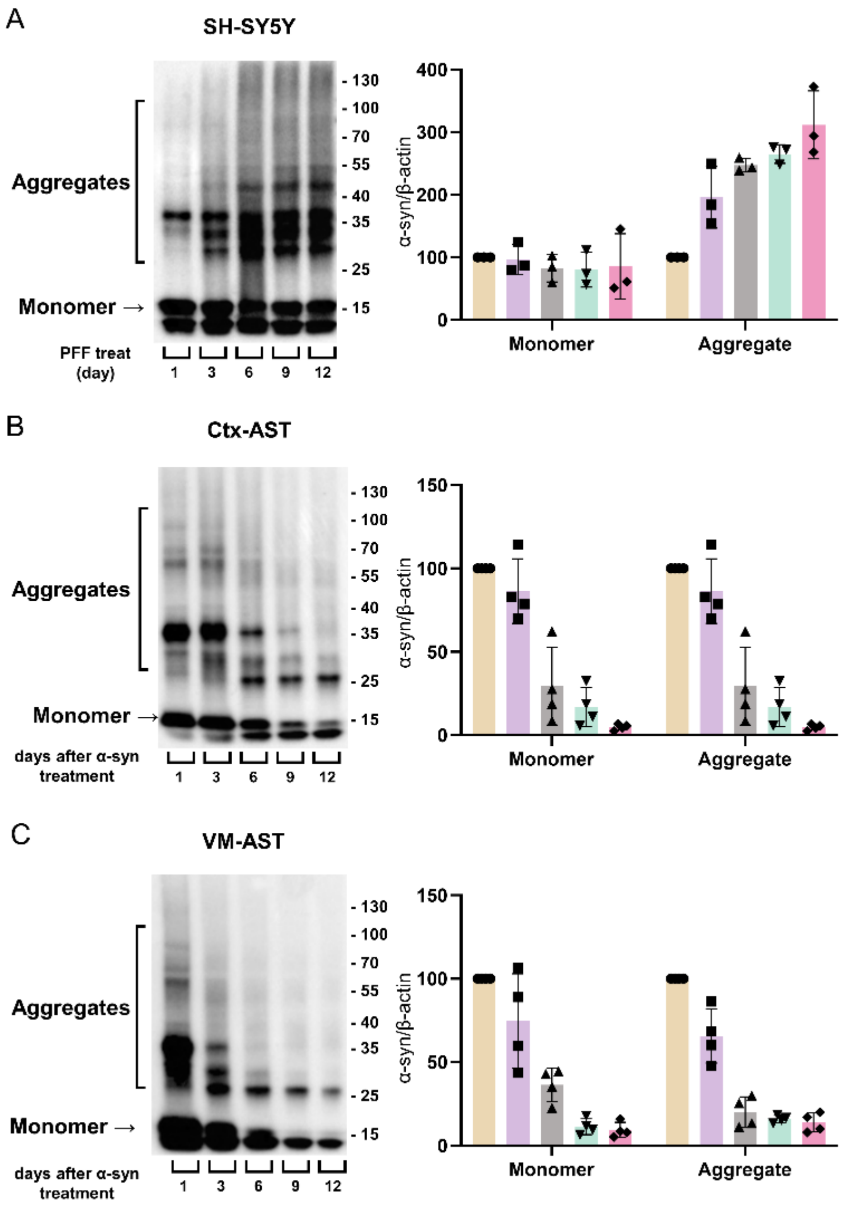

Suppl. Fig. S9. Changes of intracellular α-syn monomer and oligomer protein levels over time after exposure to α-syn-PFF. SH-SY5Y neuronal cells (**A**), Ctx-astrocytes (**B**) and VM-astrocytes (**C**) were exposed to α-syn PFF (1 μM) for 3 days. After withdrawal of α-syn PFF, intracellular protein levels of α-syn monomer and oligomers were determined using WB analysis for 12 days. Data were represented by the mean±SEM of the protein levels relative to those at day 1 (n=4).

Suppl. Fig S10

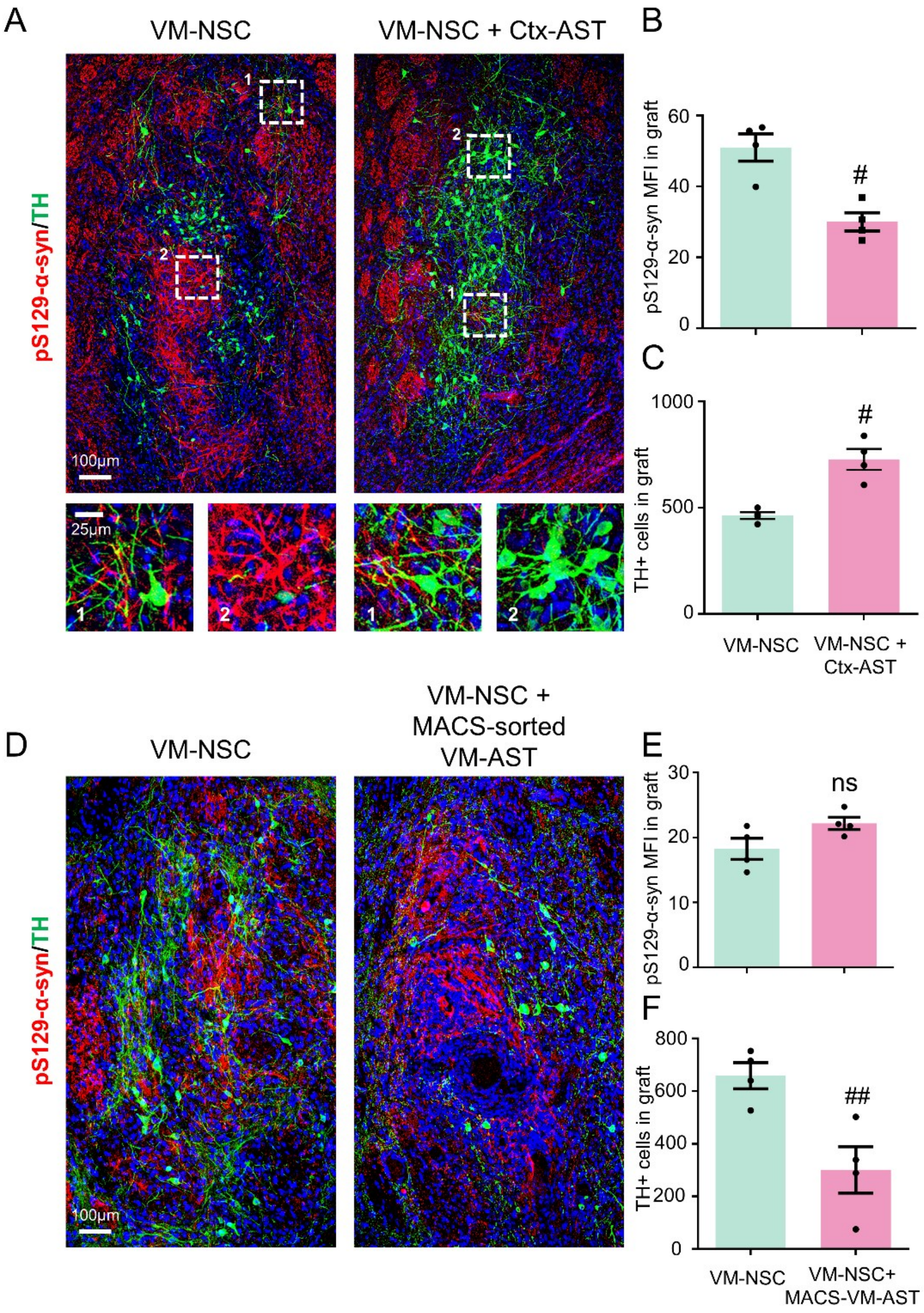

Suppl. Fig. S10. Co-grafting effects of Ctx-astrocytes (A-C) and ACSA-2-MACS-isolated astrocytes (D-F) on host-to-graft α-syn transmission and DA neuron engraftment. The intensity of pS129-αsyn immunoreactivity and the number of TH+ neurons were assessed in the grafts. Paired t-test, significant differences from the left (VM-NSC-grafted) at $p<0.05^{\#}$ and $0.01^{\#\#}$, n=4. Paired t-test.

SI References

1. Heinrich C*, et al.* (2011) Generation of subtype-specific neurons from postnatal astroglia of the mouse cerebral cortex. *Nature protocols* 6(2):214-228.
2. Song JJ*, et al.* (2018) Cografting astrocytes improves cell therapeutic outcomes in a Parkinson's disease model. *The Journal of clinical investigation* 128(1):463-482.
3. Choi YR*, et al.* (2015) FcgammaRIIB mediates the inhibitory effect of aggregated alpha-synuclein on microglial phagocytosis. *Neurobiol Dis* 83:90-99.
4. Jakobs C, Bartok E, Kubarenko A, Bauernfeind F, & Hornung V (2013) Immunoblotting for active caspase-1. *Methods Mol Biol* 1040:103-115.
5. Thakur P*, et al.* (2017) Modeling Parkinson's disease pathology by combination of fibril seeds and alpha-synuclein overexpression in the rat brain. *Proceedings of the National Academy of Sciences of the United States of America* 114(39):E8284-E8293.
